# Deletion of the *Pichia pastoris KU70* Homologue Facilitates Platform Strain Generation for Gene Expression and Synthetic Biology

**DOI:** 10.1371/journal.pone.0039720

**Published:** 2012-06-29

**Authors:** Laura Näätsaari, Beate Mistlberger, Claudia Ruth, Tanja Hajek, Franz S. Hartner, Anton Glieder

**Affiliations:** 1 Institute of Molecular Biotechnology, Graz University of Technology, Graz, Austria; 2 Austrian Centre of Industrial Biotechnology (ACIB GmbH), Graz, Austria; University of Geneva, Switzerland

## Abstract

Targeted gene replacement to generate knock-outs and knock-ins is a commonly used method to study the function of unknown genes. In the methylotrophic yeast *Pichia pastoris*, the importance of specific gene targeting has increased since the genome sequencing projects of the most commonly used strains have been accomplished, but rapid progress in the field has been impeded by inefficient mechanisms for accurate integration. To improve gene targeting efficiency in *P. pastoris*, we identified and deleted the *P. pastoris KU70* homologue. We observed a substantial increase in the targeting efficiency using the two commonly known and used integration loci *HIS4* and *ADE1*, reaching over 90% targeting efficiencies with only 250-bp flanking homologous DNA. Although the *ku70* deletion strain was noted to be more sensitive to UV rays than the corresponding wild-type strain, no lethality, severe growth retardation or loss of gene copy numbers could be detected during repetitive rounds of cultivation and induction of heterologous protein production. Furthermore, we demonstrated the use of the *ku70* deletion strain for fast and simple screening of genes in the search of new auxotrophic markers by targeting dihydroxyacetone synthase and glycerol kinase genes. Precise knock-out strains for the well-known *P. pastoris AOX1*, *ARG4* and *HIS4* genes and a whole series of expression vectors were generated based on the wild-type platform strain, providing a broad spectrum of precise tools for both intracellular and secreted production of heterologous proteins utilizing various selection markers and integration strategies for targeted or random integration of single and multiple genes. The simplicity of targeted integration in the *ku70* deletion strain will further support protein production strain generation and synthetic biology using *P. pastoris* strains as platform hosts.

## Introduction

The methylotrophic yeast *Komagataella pastoris*, commonly known as *Pichia pastoris* has become one of the major eukaryotic hosts for recombinant protein production, mainly because of its strong and tightly regulated *AOX1* promoter [1], ease of manipulation, growth to high cell-densities in inexpensive media and ability to perform complex post-translational modifications [2]. The genome sequence of the *P. pastoris* histidine auxotrophic variant GS115 has been published with a curated annotation for its 5313 protein coding genes [3]. This most commonly used and commercially available strain had been derived by mutagenesis of the *P. pastoris* WT strain NRRL-Y11430 (ATCC 76273), which was also deposited in the Netherlands as *P. pastoris* CBS7435. More recently, the nuclear and mitochondrial genome sequences of the unmodified WT strain CBS7435 were published [4]. Compared to previous sequences, most gaps were closed in the new genome sequence and some sequence and annotation mistakes were corrected. This new information shed light on the formerly poorly known, but important pathways that are responsible for methanol utilization, secretion, glycosylation, proteolytic processing and protein folding. Therefore, this information has increased the potential of *P. pastoris* and enabled its further development towards a customized and highly efficient host for heterologous protein production.

Due to the lack of stable plasmid systems for *P. pastoris*, gene expression cassettes are usually integrated into the genome. Stable integration of entire expression cassettes into the *P. pastoris* genome is based on homologous recombination (HR) and non-homologous end joining (NHEJ). HR dominant in *Saccharomyces cerevisiae* is an accurate pathway that repairs double-strand breaks (DSBs) by using the information from homologous sequences [5], [6]. The far less specific process of NHEJ, more dominant in filamentous fungi and higher eukaryotic organisms, is not necessarily accurate and deletions of a few nucleotides are often introduced at DSB-sites [6]–[11]. Gene replacement events in *P. pastoris* have been estimated to occur with a frequency of <0.1% when the total length of targeting fragments is <500 bp [12] and with a frequency of 10–20% [13] or up to 30% [12] when extensive ∼1 kb regions of homology are used. However, in previous projects we have observed that success also depends on the genomic locus. NHEJ seems to have a substantial role in the integration events of expression cassettes with flanking homologous sequences and thus limits the efficient generation of specific changes like targeted knock-outs.

**Figure 1:**
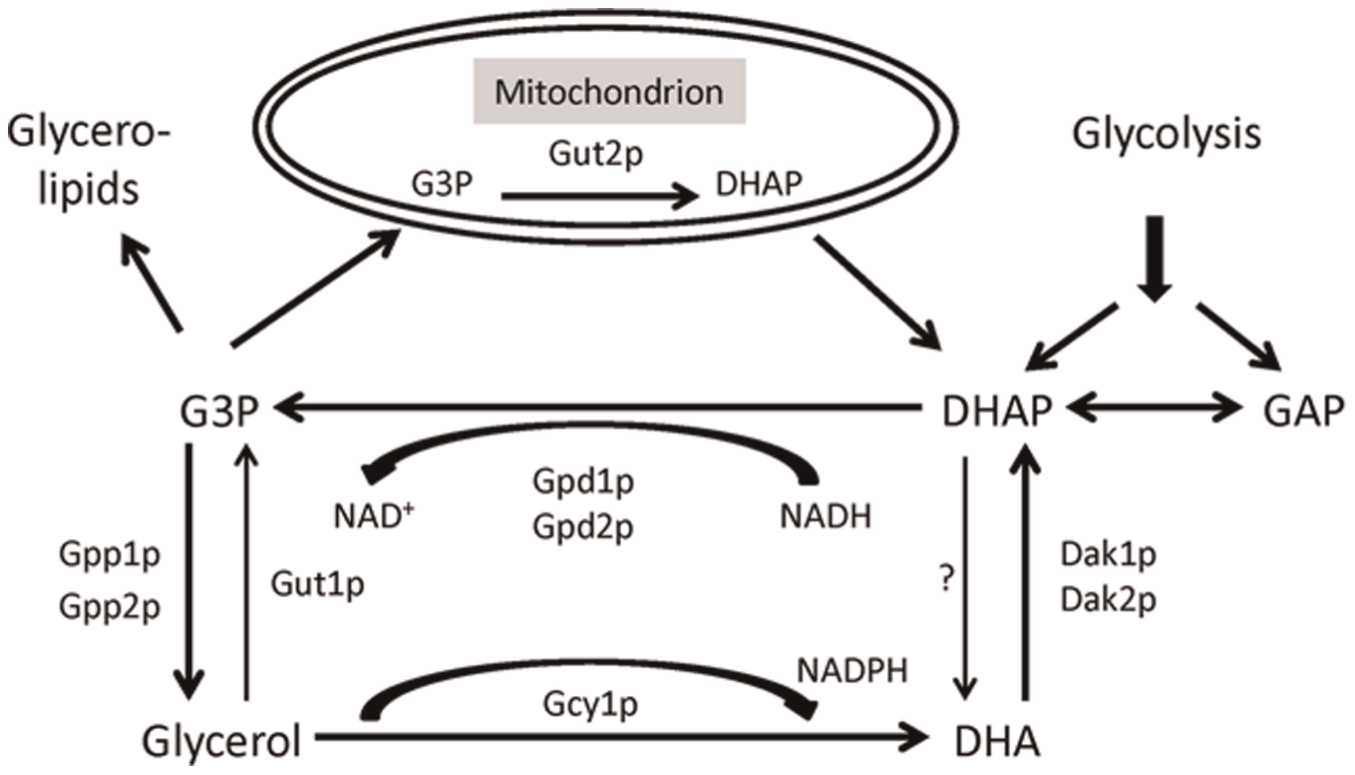
A simplified scheme of the glycerol assimilation pathway in yeast. Glycerol kinase 1 (Gut1p) knocked out in this study is involved in the first step of glycerol metabolism. Gpp1p and Gpp2p: glycerol-3-phosphatases 1 and 2, Gpd1p and Gpd2p: glycerol 3-phosphate dehydrogenases 1 and 2, Dak1 and Dak2p: dihydroxyacetone kinases 1 and 2, Gcy1p: putative NADP^(+)^ coupled glycerol dehydrogenase, DHA: dihydroxyacetone, DHAP: dihydroxyacetone phosphate, G3P: Glycerol-3-phosphate, “?”: the enzyme responsible for the conversion of DHAP to DHA is not verified. Under aerobic conditions, mitochondrial Gut2p (glycerol-3-phosphate dehydrogenase 2) can convert G3P to DHAP. Figure modified from [59].

In NHEJ, double-strand breaks are recognized by the highly conserved and arguably process defining Ku70p/Ku80p heterodimer, which specifically binds to DNA ends and forms a complex with the DNA-dependent protein kinase catalytic subunit [14]–[17]. This protein kinase, in a complex with a co-factor-like protein Xrcc4 and other auxiliary factors is known to stimulate end processing, followed by ligation by DNA ligase IV [7], [18]. Studies in both yeasts and filamentous fungi revealed that by deleting components of the NHEJ-pathway, the random integration of DNA fragments is strongly reduced [19], [20]. Therefore, in those NHEJ-pathway defective mutants, DNA integrates mainly via the HR-pathway giving rise to high homologous recombination frequencies. *Mus musculus* and *S. cerevisiae* cells that are deficient in the Ku80p counterpart of the Ku70p/Ku80p heterodimer have been reported to display telomeric shortening, be radiosensitive and exhibit V(D)J recombination defects and high levels of chromosomal aberrations [21]–[25]. However, opposite results also exist [26] suggesting that negative effects of deletions in the NHEJ pathway could be dependent on specific species and culture conditions used. In *S. cerevisiae*, mutations in the Ku70p counterpart of the Ku70p/Ku80p heterodimer have been shown to greatly impair NHEJ without perturbing telomeric functions [27]. Publications in this research field existed regarding CHO-cells [28], fungi like *Aspergillus* sp. [29]–[32], *Magnaporthe grisea* [33], *Neurospora* sp. [9] and yeasts like *Kluyveromyces lactis* [34], *Candida glabrata* [26], and *Saccharomyces cerevisiae* [9], but there was no published information regarding *P. pastoris* to date.

The objective of this study was to show that elimination of the normal function of Ku70p, a conserved DNA end-binding protein essential for NHEJ, significantly increases the homologous recombination efficiency and thus strongly reduces the problematic random integration of DNA fragments in *P. pastoris*. In order to evaluate possible advantages of a *ku70* mutant strain for the quick identification of target genes to generate new auxotrophic strains and selection systems, we targeted three significant proteins of carbon source utilisation pathways. *Dihydroxyacetone synthase 1*, *dihydroxyacetone synthase 2* and *glycerol kinase 1* from the methanol and glycerol assimilation pathways (Figure 1) were the first targets for deletion. In addition, the well-known auxotrophy marker genes *HIS4* and *ADE1* were targeted to characterize the effects of the *KU70* deletion and to generate genetically well-defined auxotrophic strains by precise single gene deletions instead of the commonly applied random mutagenesis of the whole genome. Finally, a new and well defined *P. pastoris* expression platform was generated based on the wild-type strain CBS7435 including complementary *E. coli*/*P. pastoris* shuttle vectors for protein expression.

## Materials and Methods

### Strains and Culture Conditions

All *P. pastoris* strains used and constructed during this study are based on the wild-type strain CBS7435 (NRRL-Y11430, ATCC 76273) and described in more detail in Table 1. Recombinant DNA manipulations were performed in *E. coli* strains DH5alpha and TOP10F’ (Invitrogen Corp., Carlsbad, CA) according to standard protocols [35]. All *P. pastoris* and *E. coli* strains were cultured in standard media using chemicals and other components as previously described by [36]. Amino acids were added to 40 µg/ml (histidine) or 50 µg/ml (arginine), Ampicillin to 100 µg/ml (*E. coli*) and Zeocin™ (Invitrogen) to 100 µg/ml (*P. pastoris)* or 25 µg/ml (*E. coli*) as required. *P. pastoris* competent cells were prepared with the condensed protocol and transformations were performed by electroporation, essentially as described by [37]. Before plating out aliquots on corresponding selective media, the cells (80 µl) were allowed to regenerate for two hours at 28°C with 500 µl 1 M sorbitol and 500 µl YPD. To study the effect of knock-outs in the methanol assimilation, glycerol assimilation, purine biosynthesis and amino acid synthesis pathways, target genes were disrupted with a Zeocin™ resistance cassette surrounded by two homologous ends for HR in the target locus. All *P. pastoris* platform strains constructed during this study were generated with specific full or partial knock-out of the target gene using a flipper cassette described below and similar to the construct previously described by [38].

**Table 1:** Strains of *P. pastoris* used and constructed during this work.

**Strain**	**Genotype**		**Markers**	**Source**		**BMD2%**	**BMG1%**	**BMM0.5%**
CBS7435	wild type^a^		–	CBS^b^		0.30±0.01	0.28±0.02	0.17±0.00
mut^s^	*aox1*		–	This study		0.29±0.02	0.30±0.01	0.05±0.00
mut^s^ arg-	*aox1arg4*		–	This study		0.26±0.01	0.27±0.01	0.06±0.00
mut^s^ his-	*aox1his4*		–	This study		0.31±0.01	0.29±0.01	0.05±0.00
his-	*his4*		–	This study		0.30±0.01	0.29±0.01	0.15±0.00
ku70-	*ku70*		–	This study		0.27±0.00	0.26±0.01	0.12±0.00
mut^s^ arg- c.	*aox1arg4*::ARG4		*bla*	This study		0.32±0.01	0.25±0.01	0.03±0.00
his- c.	*his4*::HIS4		*bla*	This study		0.32±0.02	0.24±0.01	0.14±0.00
mut^s^ his- c.	*aox1 his4*::HIS4		*bla*	This study		0.31±0.01	0.23±0.04	0.01±0.00
ku70-das1-	*ku70 das1*		*Sh ble*	This study		0.17±0.01	0.17±0.04	0.04±0.01
ku70-das12-	*ku70 das1das2*		*Sh ble*	This study		0.21±0.00	0.22±0.01	0
ku70-gut-	*ku70 gut1*		*Sh ble*	This study		0.26±0.01	0	0.13±0.01
ku70-gut- c.	*ku70 gut1*::*GUT1*		–	This study		0.27±0.02	0.23±0.01	0.14±0.00
ku70-his-	*ku70 his4*		–	This study		n/d	n/d	n/d
ku70-his-	*ku70 his4*		*Sh ble*	This study		n/d	n/d	n/d
ku70-ade1-	*ku70 ade1*		*Sh ble*	This study		n/d	n/d	n/d

The growth rates reported correspond to the maximal growth rates (^h-1^) reached in minimal media during the exponential growth phase. The standard deviation reported is calculated according to the growth rates of three biological replicates. c. = complemented. BM = buffered minimal media with glucose (D), glycerol (G) or methanol (M).

a NRRL Y-11430, ATCC 76273.

b Centraalbureau voor Schimmelcultures.

### Growth Rate Studies

Growth rates of the *P. pastoris* wild-type strain CBS7435 and knock-out strains *aox1*, *aox1 arg4, his4, aox1 his4, ku70*, *ku70 gut1, das1, das1 das2* and the corresponding complemented *arg4*, *his4* and *gut1* mutant strains were defined by measuring optical density (OD_595_) of triplicate cultures during exponential growth phase. An overnight culture was inoculated with a single colony in corresponding media and diluted to a starting OD of ∼0.15. The main cultures were grown in standard conditions using 50 ml of buffered minimal media BMD2% (D-glucose), BMG1% (glycerol) or BMM0.5% (methanol) in 250 ml baffled shake flasks (28°C, 120 rpm with 50mm amplitude). Amino acids were added to 40 µg/ml (histidine) or 50 µg/ml (arginine) as required for non-complemented auxotrophic strains.

### Sequence Analysis and Optimization

Nucleotide sequence data were primarily obtained from the public database NCBI (www.ncbi.nih.gov). *P. pastoris* genes *glycerol kinase 1* (*GUT1*, FR839631 region 302996-304861), *dihydroxyacetone synthase 1* (*DAS1, FR839630* region 634689-636812), *dihydroxyacetone synthase 2* (*DAS2*, *FR839630* region 630077-632200), the trifunctional *HIS4 (phosphoribosyl-ATP pyrophosphohydrolase, phosphoribosyl-AMP cyclohydrolase, and histidinol dehydrogenase*, U14126.1), *argininosuccinate lyase 4* (*ARG4*, AF321097.1) and *KU70* (FR839630, region 1598101-1599963, XM_002492501.1) were identified either by their annotation or by a blastx search of the genome sequence of *P. pastoris* CBS7435. Other sequences of the *DAS1* and *DAS2* genes in public databases contained mistakes due to the wrong assembly of raw data caused by the high sequence similarity of these genes [4]. Excision cassette constructs and plasmids were designed using VectorNTI (Invitrogen, Carlsbad, CA, USA). Primers were designed manually and analyzed with EditSeq (DNASTAR, Madison, WI, USA). ClustalW [39] was used for the pairwise and multiple alignments of known sequences and the pI/Mw tool from ExPASy Proteomics Server was used for calculating the isoelectric points and molecular weights of proteins. To confirm the expected knock-outs and plasmid compositions, the sequences obtained from Sanger sequencing (LGC Genomics, Berlin, Germany) were assembled using SeqMan (DNASTAR). Synthetic genes were codon compromised according to a combined average codon usage of *Pichia pastoris*, *Yarrowia lipolytica* and *Schizosaccharomyces pombe* using GeneDesigner (DNA 2.0, Menlo Park, CA, USA) [40]. This strategy avoided very rare codons for any of these three hosts to provide vector elements for a broad host spectrum.

### Excision Cassettes and Gene Disruption

**Figure 2:**
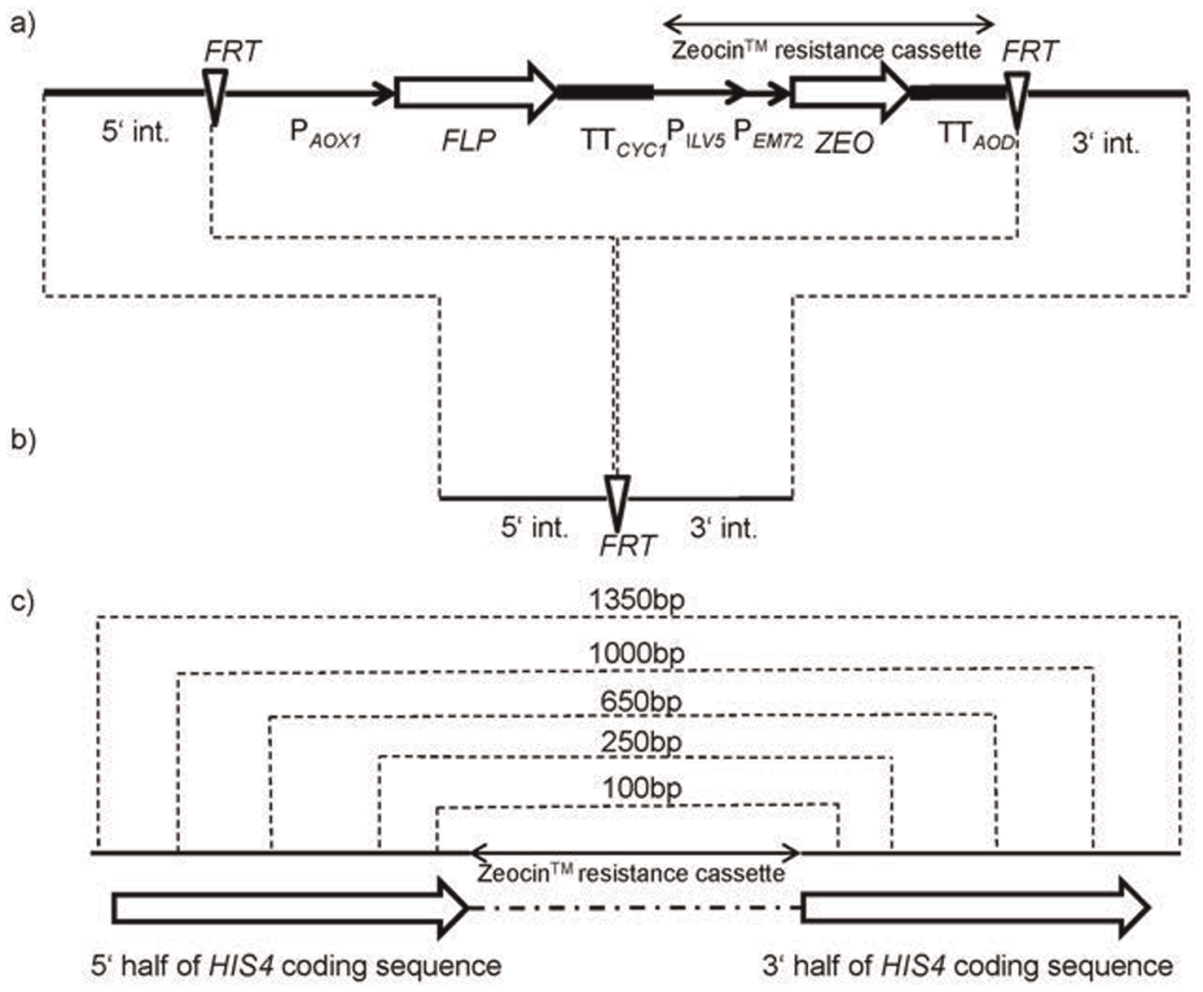
Integration cassette composition and function. a) *KU70* disruption cassette based on the *S. cerevisiae* FLP recombinase system. On both sides the flipper cassette with *AOX1* promoter (*P_AOX1_*), FLP recombinase (*FLP*), CYC1 terminator (*CYC1_TT_*) and Zeocin™ resistance cassette are surrounded by recombinase target sequences (*FRT*) and locus specific integration sequences (5′int and 3′int). Cassette components are not drawn to scale. b) After methanol induced (*P_AOX1_*) FLP production and subsequent *FRT* recognition leading to cassette excision only one *FRT* (34 bp) is left in the locus in between the 3′ and 5′ integration sequences. c) The lengths of the homologous sequences at 5′ and 3′ ends of the disruption cassettes used to compare the homologous recombination frequencies in wt and ku70 deletion strains varied from 100 bp to 1350 bp in the *HIS4* locus. Zeocin™ resistance cassette was placed in between the homologous sequences. Cassette components are not drawn to scale.

The structure of all excision cassettes used to create the new platform strains is, in principle, as described by [38], wherein the FLP recombinase system [41] is utilized to enable excision cassette removal. A marker free knock-out strain is created with one 34 bp FLP recombinase recombination target sequence (*FRT*, GAAGTTCCTATACTTTCTAGAGAATAGGAACTTC) left in the locus. All cassette components used were either amplified from wild-type strains (*P. pastoris* CBS7435, *S. cerevisiae* BY4741) or synthetic fragments. The *P. pastoris AOX1* promoter was chosen to drive the regulated expression of the *S. cerevisiae FLP* recombinase terminated by the *S. cerevisiae CYC1* terminator. A Zeocin™ resistance cassette was amplified from pPpT2 [36]. The above-mentioned parts were surrounded on both sides by identical 34 bp *FRT*s placed in direct orientation. The outermost parts of each excision cassette, namely the 5′ and 3′ integration sequences, were locus-specific to guarantee a knock-out only in the target region. All parts were amplified and joined by standard overlap-extension PCR using HPLC-purified primers (Eurogentec, Seraing, Belgium) listed in Table S1 and Phusion™ High-Fidelity DNA-polymerase (Finnzymes Oy, Espoo, Finland). For the overlap-extension PCRs, equimolar amounts of each fragment were used. In the case of the *AOX1* knock-out cassette, the *AOX1* promoter was placed 5′ of the first *FRT* to simultaneously function as a promoter for the FLP recombinase and as a 5′ integration sequence. The length of the integration sequences and the extent of the knock-outs (described in Table 2) were dependent on the sequence information available at the time of cassette design. To ensure the inactivation of the *KU70* gene, the start codon ATG was also modified to ATAC. An example of the cassette structure is illustrated in Figure 2a. Gene disruption cassettes to study both homologous recombination in the *ADE1* and *HIS4* loci of the *ku70* deletion strain and the effect of knock-outs in glycerol and methanol assimilation pathways were constructed by overlap-extension PCR joining the Zeocin™ resistance cassette with 5′ and 3′ locus-specific sequences for targeted homologous recombination (Figure 2b). The lengths of the homologous sequences used are depicted in Table 3. Figure 2b illustrates the method used to achieve gene inactivation in *HIS4* locus despite the extent of homologous integration.

**Table 2:** Excision cassette structures.

Locus	5′ integration	3′ integration	Knock-out size
***KU70***	−200 to +1099	+1315 to +2484	215 bp
***AOX1***	−939 to −1	+1993 to +3136	1992 bp
***HIS4***	−894 to −51	+2533 to +3414	2582 bp
***ARG4***	+22 to +984	+1399 to +2900	414 bp
***GK1***	−169 to +752	+1138 to +2050	385 bp
***DAS1***	+62 to +580	+581 to +1133	0 (disruption)
***DAS2***	+62 to +580	+581 to +1133	0 (disruption)

Homologous integration sequence lengths used in the 5′ and 3′ ends of the excision cassettes were defined by the structure of the target locus and the sequence data available at the time point of cassette design.

**Table 3:** Homologous recombination frequency (HRFb) in wt CBS7435 and *ku70* deletion strains.

Recipientstrain	Locus	Length of homology on each side	Auxotrophic/total number of transformants counted	HRFb%
wt	*HIS4*	∼1350 bp	44/266	16.5
		∼1000 bp	31/268	11.6
		∼650 bp	32/268	11.9
		∼250 bp	8/268	3.0
		∼100 bp	1/268	0
	*ADE1*	∼650 bp	0/>10^3^	0
		∼400 bp	0/>10^3^	0
		∼150 bp	11/>10^3^	0
		∼50 bp	0/>10^3^	0
*ku70*	*HIS4*	∼1350 bp	267/268	100
		∼1000 bp	268/268	100
		∼650 bp	267/268	100
		∼250 bp	86/89	96.6
		∼100 bp	20/57	35.1
	*ADE1*	∼650 bp	465/533	87.9
		∼400 bp	114/172	75.4
		∼150 bp	12/99	17.5
		∼50 bp	1/317	0

Over 95% homologous recombination frequencies could be reached in the *ku70* deletion strain with as little as 250 bp of homologous sequence on each side of the integration cassette. In the corresponding wild-type strain, only 16.5% homologous recombination frequency was reached with the longest (1350 bp) homologous sequence tested.

The overlap-extension PCR products were purified using Wizard® SV Gel and PCR Clean-Up System (Promega, Madison, WI, USA) and cloned into the pJET vector using CloneJET kit (Fermentas). The correct cassette structure was verified by sequencing followed by cassette amplification, purification and transformation into appropriate *P. pastoris* strains. Only a low amount of DNA (∼500 ng/80 µl cells) was used to guarantee the prevalence of single-copy integration. Positive transformants were grown in 96-well deep-well plates [42] for 2.5 days and pinned onto either minimal methanol (*AOX1* knock-out) or minimal dextrose (*ARG4* and *HIS4* knock-outs) agar plates to distinguish between the strains with normal and slow-growth/no-growth phenotypes. The frequency of correct integration into the *ADE1* locus was defined by observing the transformation plates and calculating red colonies resulting from the accumulation of biosynthetic intermediate phosphoribosyl aminoimidazole. Due to a significant difference in the growth rates of the *ade1* and wild-type strains, the plating out of transformants was performed only after significant dilution, with the aim of observing approximately 10 colonies on each plate. Methanol induction to initiate the production of the FLP recombinase and thus achieve cassette excision was performed by two consecutive rounds of cultivation on minimal methanol plates. After a total of 5 days, single colonies were picked from the methanol plates and streaked out on YPD- Zeocin™ plates to identify the clones that had excised the cassette and were Zeocin™-sensitive.

### Plasmid Constructions

The sequences of the plasmids in our *P. pastoris* expression platform were deposited in the GenBank database at NCBI. The plasmids pPpB1_S (JQ519685), pPpB1GAP (JQ519686), pPpB1GAP_S (JQ519687), pPpB1_Alpha_S (JQ519688), pPpT4 (JQ519689), pPpT4_S (JQ519690), pPpT4_Alpha_S (JQ519691), pPpT4GAP_S (JQ519692), pPpT4GAP_Alpha_S (JQ519693), pPpKan_S (JQ519694) and pPpKan_Alpha_S (JQ519695) were constructed as described previously [36], [43], [44]. Primers and the components and origins of components used in the plasmid construction are described in Tables S1 and S2. The vector maps are elucidated in Figure S1.

The complementation plasmids pPpARG4 (JQ519696) and pPpHIS4 (JQ519697) were constructed as follows. The codon usages of the coding sequences (cds) of both synthetic marker genes were designed to be the combined average codon usage of *P. pastoris*, *Yarrowia lipolytica* and *Schizosaccharomyces pombe.* Codons with an appearance of less than 8% in any of the above organisms were excluded. The expression unit, including the *AOX1* promoter, multiple cloning site and *AOX1* terminator, was amplified from pPpT2 [36]. The *BLA* cds and pUC origin of replication were amplified from pUC8. The synthetic prokaryotic *EM72* promoter was constructed using HPLC purified primers. For pPpARG4, the natural promoter and terminator sequences were amplified from the wt genomic DNA. For the plasmid pPpHIS4, *ADH1* promoter and *TIF51A* terminator were chosen to be used. Both parts were amplified from *S. cerevisiae* wt genomic DNA. All parts were joined by overlap-extension PCR before the linear cassette was digested by XbaI (Fermentas) and circularized using T4 ligase (Fermentas).

A glycerol kinase complementation plasmid pPpGUT1 (JQ519698) was constructed using both pPpT2 for *AOX1* promoter driven expression and a 3275 bp fragment from the glycerol kinase locus that included the natural promoter, cds and terminator for selection in the *P. pastoris gut1* deletion strain. This vector was designed as a replacement vector and therefore contained 5′and 3′homologous regions of the *GUT1* locus for site specific integration and replacement of the antibiotic marker in the *gut1* deletion strain.

### Methods Used in the Characterization of the Strains and Plasmids

Genomic DNA was isolated from each strain using DNAeasy kit (Invitrogen Corp., Carlsbad, CA) according to the manufacturer’s protocol for large scale yeast DNA isolation. All targeted loci in the *P. pastoris* genome were PCR amplified and sequenced to confirm expected knock-outs using primers described in Table S1 and Phusion™ High-Fidelity DNA-polymerase according to manufacturer’s recommendations. Southern blotting and hybridization were carried out according to standard protocols [45] to verify the correct integration of the excision cassettes. PCR amplification of the probes specific to the Zeocin™ gene and knock-out regions in genes *AOX1*, *HIS4* and *KU70* was performed using DIG-labeled dNTPs (Roche, Basel, Switzerland) and AmpliTaqGOLD® (Roche, Basel, Switzerland) polymerase according to the manufacturers’ instructions. Primer details can be found in Table S1.

Correct plasmid sequences were confirmed by Sanger sequencing. For testing the functionality, an improved version of green fluorescent protein [46] was cloned into the multiple cloning site of each plasmid. Correct insertion was verified by sequencing. Each plasmid was amplified in *E. coli*, linearized with BglII or SmiI (Fermentas) and transformed into the corresponding *P. pastoris* strains. Single colonies were transferred to 96-well deep-well plates for standard cultivation as described previously [42]. To compare the expression levels, GFP fluorescence was measured as described by [47].

### Genome Walking Experiments

In addition to the expected locus, gene disruption cassettes might also integrate at other loci of the genome. Genome walking was used to define the integration sites in strains where targeting of the cassettes seemed to be random. Therefore 2 µg of genomic DNA of each strain was singly digested with BamHI (a), EcoRI (b) and HindIII (c) in order to get fragments of 1 – 5 kb size. The digestion was stopped by heat inactivation, the fragmented DNA precipitated with ethanol and the pellet dissolved in 30 µl of distilled water.

An adaptor fragment was created by annealing adaptor strand 1 (5′-GTAATACGACTCACTATAGGGCACGCGTGGTCGACGGCCCGGGCTGGT-3′) either to adaptor strand 2.a (3′-TCCCCGACCACTAG-5′) for BamHI digested DNA, 2.b (3′-TCCCCGACCATTAA-5′) for EcoRI digested DNA or 2.c (3′-TCCCCGACCATCGA-5′) for HindIII digested DNA.

In the annealing reaction, adaptor strand 1 (100 µM, 13,7 µl) was mixed in a 1∶1 molar ratio with adaptor strand 2.a, 2.b or 2.c (100 µM, 4 µl) and denatured in 95°C for 5 min. The mixture was allowed to slowly cool down to room temperature. The three differently annealed adaptors 1+2a (BamHI), 1+2b (EcoRI) and 1+2c (HindIII) were ligated for 3 h at room temperature with T4 DNA ligase to the digested DNA fragments, considering the specific 5′ overhangs that had been created by the restriction enzymes used. The ligation reaction was stopped by incubation for 5 min at 70°C and diluted with 70 µl TE buffer.

Two gene-specific primers and two adaptor primers were designed (Table S1). The gene-specific primers were designed to bind approximately 100 bp away from the end of the known sequence, considering that no restriction site of the restriction enzymes used for gDNA digestion lay between the primer-binding site and the end of the known sequence. Adaptor primer 1 and gene specific primer 1 were used as a primer pair for a first PCR with 1 µl of the gDNA-adaptor ligation product as template DNA. 1 µl of the first PCR mix was used as the template for a second PCR with adaptor primer 2 and gene specific primer 2. This second primer pair was designed to bind within the first PCR product. Both PCR steps were performed with an annealing temperature of 58°C and an elongation time of 50 seconds. A gene-specific DNA fragment as a product from the second PCR was isolated from a preparative agarose gel, purified and sent to Sanger sequencing (LGC Genomics GmbH, Berlin, Germany) using adaptor primer 2 and the corresponding gene specific primer 2.

### Genetic Stability Tests

To test the stability of multiple expression cassettes in the wild-type and *ku70* deletion strains during repetitive phases of growth on glucose and methanol, the wt strain CBS7435 and deletion strains CBS7435 mut^s^ and CBS7435 *ku70* were transformed with 3 µg of SmiI- linearized plasmid pPpT4_S_GFP. The resulting colonies were screened for GFP activity as described by [47] and the best expressing clones were preserved in glycerol stocks. Duplicates of each strain were inoculated to a starting OD of 0.55 in 50 ml BMD1% in 250 ml baffled shaking flasks. The strains were cultivated for 48 h to reach a comparable cell density (28°C, 120 rpm with 25 mm amplitude), followed by a methanol induction phase for 72 h [42]. The growth and induction cycles were repeated for four times, always using the induced culture as a starting material for the next round. DNA was extracted from the starting strains and the strains after four rounds of methanol induction as described previously [48]. Copy numbers were detected with qRT-PCR as described by [44]. Each qRT-PCR was performed at least twice by independent researchers. Strains displaying a standard deviation of over one copy number between independent duplicates were omitted from the analysis.

### Spread Titer Tests

To measure the sensitivity of the *ku70* deletion strain to UV light, a spread titer test was performed as follows. Overnight cultures of four biological replicates of each wt and *ku70* deletion strain cells grown in YPD were harvested and washed. The OD was adjusted to 1 prior to plating out 50 µl and 100 µl aliquots of 10^−4^ and 10^−5^ dilutions respectively on YPD agar. The plates were exposed to 0, 50 and 100 J/m^2^ UV rays (Bio-Link 254 UV crosslinker, Vilber Lourmat, Marne-la-Vallée Cedex, France), and incubated in the dark for 60 h before calculating the amount of colony forming units on exposed and unexposed plates.

## Results

### Identification of the Target Genes *KU70*, *KU80*, *GUT1*, *DAS1* and *DAS2* in the Wild-type *P. pastoris* CBS7435 Genome

Non-homologous end-joining is one of the main pathways repairing broken genomic DNA in eukaryotes. One of the aims of our study was to investigate if the NHEJ pathway plays an important role in the site targeted specific integration in the genome of *P. pastoris* (NRRL-Y 11430, ATCC 76273) and if the NHEJ mechanism can be impaired by knock-out mutagenesis. Homologues to the nucleotide sequences of the *S. cerevisiae* genes *HDF1* (NM_001182791.1) and *HDF2* (NM_001182606.1), corresponding to *KU70* and *KU80* respectively, were identified in the *P. pastoris* wild-type genome by blastx. The *KU70* homologue (FR839630, region 1598101-1599963) in chromosome 3 shared 28% sequence identity to *HDF1* and the *KU80* homologue (FR839631.1 Region 361953-362288, 362551-364041) in chromosome 4 showed 19% sequence identity to *HDF2* (both defined by pairwise alignment using ClustalW). Both genes had been annotated as subunits of the telomeric Ku complex (yeast Ku70p/Ku80p) by the automated annotation of the assembled genome. Remarkably, the sizes of the Ku70 and Ku80 proteins, compared to the corresponding proteins of *S. cerevisiae, H. sapiens* and *A. thaliana*, would better cohere with contrary annotation. The *P. pastoris KU70* and *KU80* cDNAs encode proteins of 620 and 608 amino acids with predicted molecular masses of 71.3 and 69.5kD. The protein sizes are similar to those of *S. cerevisiae* (602aa and 629aa, 70.6 and 71.2kDa), *H. sapiens* (P12956 609aa and P13010 732aa, 69.8 and 82.7kDa) and *A. thaliana* (Q9FQ08 621aa and Q9FQ09 680aa, 70.3 and 76.7kDa). However, the knock-out of either subunit has been reported to result in reciprocal down-regulation of the other subunit [49], thus making a confirmation of correct annotation by function troublesome. The sequence identities to already known Ku proteins are generally low. The *P. pastoris* Ku70 and Ku80 proteins share only 28% & 19%, 18% & 16%, and 15% & 16% sequence identity with the proteins of *S. cerevisiae*, *H. sapiens* and *A. thaliana,* respectively.

For a first feasibility test to generate well defined auxotrophic strains based on the *P. pastoris* wt strain CBS7435, the nucleotide sequences of the three genes g*lycerol kinase* 1 (*GUT1*, FR839631 region 302996-304861, 621aa), *dihydroxyacetone synthase 1* (*DAS1, FR839630* region 634689-636812, 707aa) and *dihydroxyacetone synthase 2* (*DAS2*, *FR839630* region 630077-632200, 707aa) from the glycerol and methanol assimilation pathways were elucidated.

### Construction and Molecular Characterization of a *P.* *pastoris ku70* Deletion Strain

The *KU70* flipper knock-out cassette was constructed with 1299bp 5′ and 1170bp 3′ flanking sequences from the *KU70* locus. Both integration sequences were placed partially on top of the coding sequence, so that the deletion caused by the knock-out cassette integration would be only 215bp. This short deletion leading to inactivation of the Ku70 protein is optimal for complementation of the locus if needed. The 6747 bp assembled linear PCR product was successfully used to transform the *P. pastoris* wild-type strain CBS7435. Control PCRs were performed to confirm correct cassette integration, excision and expected knock-out in the cds of *KU70*. In the first PCR reaction, both primers (Table S1) were located outside the target region. This reaction functioned as a positive control, PCR amplifying ∼2.7 kb, ∼2.5 kb and ∼6.7 kb fragments from the wt, the *ku70* deletion strain after flipper excision and the *ku70* deletion strain before flipper excision. The second primer pair served as a negative control PCR, since one of the primers bound in the region knocked out in *ku70* deletion strains. A ∼1.3 kb product was expected from the wt strain and any other strain having the excision cassette integrated somewhere other than in the *KU70* locus of the genome. One strain giving expected PCR product sizes from all control PCRs was chosen for further tests. High quality genomic DNA was isolated from the wt starting strain, the original transformant strain before flipper excision and the final *ku70* deletion strain after flipper excision. The purified genomic DNA was subjected to southern blot analysis after two separate digestions with restriction enzymes BamHI and BglII. When using a probe specific to the knock-out region in the *KU70* locus, hybridizing fragments of expected sizes (∼4.4 kb for BamHI and ∼4.1 kb for BglII) were observed in the wild-type strain. Fragments were missing in both *ku70* deletion strains (before and after cassette excision) verifying correct cassette integration and successful knock-out. To rule out knock-outs elsewhere in the genome, single-copy integration of the excision cassette in the expected locus was tested by hybridizing a probe specific to the Zeocin™ resistance cassette to both BamHI and BglII digested genomic DNA of the before mentioned strains. Fragments of the expected size (8.4 kb BamHI, 4.8 kb BglII) could be observed in the strain still carrying the resistance cassette before induction, suggesting single-copy integration in the expected locus. No bands were observed either from the wt strain or *ku70* knock-out strains (Figure S2). Furthermore a 1.4 kb fragment of the deletion site was amplified from the genomic DNA of the *ku70* knock-out strain and sequenced. This sequence (Figure S3) verified a correct gene deletion and the expected FRT sequence left in the locus.

The behaviour of the *ku70* deletion strain in heterologous protein production was tested with SmiI linearized pPpT4_GFP plasmid in comparison to the corresponding wt strain. The fluorescence intensity landscapes resulting from the screening of two 96-well plates per strain were noted to be more uniform for the *ku70* deletion strain (data not shown). No increased amount of inactive clones could be detected.

### Increased Targeting Efficiency Employing the *ku70* Deletion Strain

To compare the gene targeting efficiency of the *ku70* knock-out and wt strains, the loci of two biosynthetic genes *ADE1* and *HIS4* were chosen due to the reliable and simple detection of corresponding auxotrophies. *P. pastoris* colonies lacking *ADE1* develop red color when grown without adenine supplementation and *his4* knock-out colonies can be detected by replica plating on minimal media without histidine supplementation.

The excision cassettes to disrupt *ADE1* and *HIS4* genes were successfully constructed by overlap-extension PCR as described previously. The cassettes were planned to disrupt the target gene despite the fact that the excision cassette might recombine using either 5′ or 3′ integration sequence only. Increasing the length of homologous sequences has been reported to have a positive effect on the homologous recombination frequency in *N. crassa* [19]. 1 kbp has been suggested to be the minimum length of 5′ and 3′ homologous regions required for 100% and >85% site-specific recombination and replacement of target genes in *ku70* deletion mutants of *N. crassa* and *S. macrospora* respectively [19], [20]. Also, in *P. pastoris*, longer homologous sequences are important for efficient integration into the genome [12]. Therefore, we chose homologous flanking sequence lengths varying from 1350 bp down to only 100 bp to examine the recombination frequency in the *HIS4* locus of *P. pastoris*. The same study was done for the *ADE1* locus. However, since the homologous recombination frequencies for the *ku70* deletion strain with flanking sequence lengths of 1350 bp down to 650 bp were still about 100% in the *HIS4* locus, a set of shorter flanking sequences varying from 650 bp to 50 bp was chosen to point out strain specific differences for the *ADE1* locus. The homologous recombination frequencies in the wt and *ku70* knock-out strains are depicted in Table 3. In contrast to the wt strain, almost all antibiotic resistant transformants of the *ku70* deletion strain showed the expected phenotype – even when short homologous sequences of 150–250 bases were used for integration. This observation indicated correct site-specific integration for the majority of the *ku70* knock-out strain transformants whereas most of the constructs integrated at different sites in the case of the wt strain. Homologous recombination efficiency in the *ku70* deletion strain dropped to the level of the wt with integration sequence lengths as short as 50–100 bp. The potential artefacts caused by variations between transformations and radically different growth rates of wt and *ade1* strains were minimized by repeating every transformation for four times and plating small aliquots of diluted cells. However, the possibility of wt colonies overgrowing *ade1* colonies and thus affecting the calculation of homologous recombination frequencies cannot be ruled out, especially for the wt host strain with almost no homologous recombination in the *ADE1* locus.

A majority of the cassettes targeted to either *HIS4* or *ADE1* loci of the wild-type strain did not result in auxotrophy. Therefore, the integration loci of altogether 18 wt and mut^s^ strains were analysed by genome walking in order to verify the assumption that integration happens randomly in these strains. Although very low amounts of DNA were used for transformation, and thus most of the colonies were expected to be single-copy transformants, multi-copy integration could not be ruled out. Therefore only 14 strains delivering a strong main product in the nested PCR of genome walking and high sequence quality were included in the final analysis depicted in Table 4. In addition, the integration loci of four strains were verified by digestion with two different restriction enzymes.

**Table 4:** Selection marker integration.

Strain #	Digestion	Strain	Integration sequence	Integration locus	Gene hit
1	HindIII	wt	HIS4 250 bp	Chr. 2 (1088794)	Protein Mis14
1	EcoRI	wt	HIS4 250 bp	Chr. 2 (1088794)	Protein Mis14
2	BglII	wt	HIS4 250 bp	Chr. 4 (1549929)	Cell morphogenesis protein Pag1
3	BamHI	mut^s^	HIS4 250 bp	Chr. 1 (2815628)	Non-coding: 64 bp at 5′ side: Protein Tos1, 292 bp at 3′ side: Uncharacterized protein YPL066W
3	BglII	mut^s^	HIS4 250 bp	Chr. 1 (2815628)	Non-coding: 64 bp at 5′ side: Protein Tos1, 292 bp at 3′ side: Uncharacterized protein YPL066W
4	BglII	mut^s^	HIS4 250 bp	Chr. 4 (1356618)	Non-coding: upstream of hypothetical protein
6	HindIII	wt	HIS4 250bp	Chr. 1 (1935839)	Non-coding: 98 bp at 5′ side: Protein Lst4, 310 bp at 3′ side: protein midasin
8	BamHI	mut^s^	HIS4 250 bp	Chr. 4 (1549929)	Cell morphogenesis protein Pag1
10	BglII	wt	HIS4 250 bp	Chr. 3 (1866738)	Non-coding: 66 bp at 5′ side: Zinc finger protein 167, 288 bp at 3′ side: 1,3-beta-glucanosyltransferase
10	BamHI	wt	HIS4 250 bp	Chr. 3 (1866738)	Non-coding: 66 bp at 5′ side: Zinc finger protein 167, 288 bp at 3′ side: 1,3-beta-glucanosyltransferase
11	HindIII	mut^s^	HIS4 250 bp	Chr. 4 (1354273)	Protein Ecm3
12	BamHI	mut^s^	HIS4 250 bp	Chr. 1 (2137406)	Ammonium transporter protein Mep2
13	HindIII	wt	HIS4 250 bp	Chr. 1 (2706933)	Inositol 2-dehydrogenase
14	BamHI	mut^s^	HIS4 250 bp	Chr. 4 (846891)	Non-coding: 188 bp at 5′ side: Protein Letm1 and EF-hand domain-containing protein anon-60Da, 143 bp at 3′ side: phosphatidylinositol 3-kinase
19	EcoRI	wt	ADE1 150 bp	Chr. 3 (87007)	Non-coding: 420 bp at 3′ side: mannose-6-phosphate isomerase
20	BamHI	wt	ADE1 150 bp	Chr. 1 (1994439)	Likely SIR2 family histone deacetylase
21	EcoRI	wt	ADE1 150 bp	Chr. 3 (2074903)	Eukaryotic translation initiation factor 2 subunit alpha
21	HindIII	wt	ADE1 150 bp	Chr. 3 (2074903)	Eukaryotic translation initiation factor 2 subunit alpha

Integration sites of the gene disruption cassettes in *P. pastoris* wt strains which remained autotroph after selection marker integration.

Cassette integration was detected in every chromosome and, for most of the strains, seemed to be random. No micro-homologies (∼10 bp) at the exact site of integration were detected by sequencing the 5′ end of the disruption cassette. Interestingly, the integration loci of the strains #4 and #11 in chromosome 4 were separated by only ∼3.5 kbp. In addition, the integration locus of strains #2 and #8 in chromosome 4 was identical. No explanation of these facts could be detected by sequence analysis. However, the identical integration locus of strains #2 and #8 could also be explained by a possible clone identity due to duplication of the transformant during the regeneration phase after electroporation. After transformation, the cells were regenerated in sorbitol/YPD for approximately 2 hours, which theoretically allows one cell division.

In NHEJ, the possibility of inaccuracy and introduction of small deletions at the DSB-sites has been reported [6]–[11]. Also, in two of the 14 strains analyzed in this study, a single nucleotide deletion in the 5′ end of the disruption cassette was detected. However, considering the fact that only 5′ sequences were determined by genome walking and the relatively low number of analyzed random integrants, no reliable determination of the total frequency of deletions at the DSB sites can be made.

### Application of the *P. pastoris ku70* Deletion Strain as a Tool to Screen Targets for the Generation of Auxotrophic Strains

Low rates of site-specific integration in the expected gene locus make it difficult to study the effect of site-specific integration or knock-out variants in the wt strains. Many transformants have to be analyzed on a molecular level (e.g. by colony PCR, sequencing, southern blot) since many of the selective markers integrate at other sites. For hard to access loci most of the transformants show integration of the selection marker at different sites (as for *HIS4* and *ADE1* described above), which can lead to the misinterpretation that the targeted gene might be essential. The use of the *P. pastoris ku70* deletion strain offers a possibility to reduce the number of positive transformants showing integration of the selection marker without disrupting the target gene and therefore facilitates and speeds up knock-out strain identification. After quick identification of a suitable locus, this can still be repeated with the wt strain or alternatively the *KU70* deletion can be complemented again. Since all clones where the *HIS4* and *ADE1* loci were targeted displayed the expected auxotrophic phenotype, we took advantage of the high rate of site-specific integration in the *ku70 deletion* strain for a quick and simple evaluation of the feasibility for a few selected genes to serve as targets to generate new auxotrophic strains.

For the search of possible new *P. pastoris* auxotrophy strains, knock-out cassettes to disrupt *GUT1*, *DAS1* and *DAS2* were constructed and used for successful transformation of the *P. pastoris ku70* knock-out strain. Since the knock-out cassettes contain long (500–1000 bp) homologous integration sequences, all eight clones tested had the cassette integrated in the right locus, resulting in the expected gene disruptions. Specific inactivation (partial replacement) of the *glycerol kinase (GUT1)* gene resulted in a *P. pastoris* strain that was observed to have abolished growth on glycerol as the sole carbon source. Initial experiments proved the possibility of complementing the auxotrophy with plasmid pPpGUT1 (Table 1) and of selecting corresponding transformants on minimal glycerol medium. Even though a disruption of the genes *DAS1* and *DAS2* lead to phenotypes with reduced growth on methanol (Table 1), the ability to grow on minimal media agar plates with methanol as the sole carbon source was not totally abolished, leading to too high background growth for use as a selection marker. We speculate that another enzyme, like transketolase *TKL1*, with similarity to the known dihydroxyacetone synthases could, to some extent, take over their role in the peroxisomes.

### Comparative Genetic Stability of Transformants

According to [50], the genetic stability of *P. pastoris,* strains carrying multiple (≥12) copies of an expression cassette is dependent on conditions which induce target gene expression. However, low-copy transformants (1–6 copies) were shown to exhibit high stability regardless of whether induced or not. To rule out that changes in the normal repair mechanisms of the *ku70* deletion strain could cause significantly increased genetic instability of expression strains, a set of *P. pastoris* transformants carrying four to seven copies of P*_AOX_*-GFP (plasmid pPpT4_S) were employed to investigate the genetic stability of the *ku70* deletion strains during methanol induction. No changes in the copy numbers could be detected even after four 72 h rounds of methanol induction, corresponding to a total cultivation time of 480 h and a total induction time of 288 h (over 100 generations) in non-selective media (the results are described in Table S3).

### Susceptibility of the *ku70* Deletion Strain to DNA Damage Induced by UV Light

The Ku70p/Ku80p heterodimer has been suggested to be involved in DNA-repair processes [14]. To measure the sensitivity of the *P. pastoris ku70* deletion strain to UV light, a spread titer test was performed with four biological replicates and 0, 50 and 100 J/m^2^ exposure. The survival rates determined by calculating the amount of colony forming units after 60 h incubation were 58% (±6,9%) for the wt strain and 48% (±6,8%) for the *ku70* deletion strain with 50 J/m^2^ exposure. The corresponding values for wt and *ku70* deletion strains when using an exposure of 100 J/m^2^ were 37% (±5,6%) and 27% (±5,6%), respectively, confirming the expected reduced survival rate.

### New *P. pastoris* mut^S^, *arg4* Deletion and *his4* Deletion Platform Strains and Complementary *E. coli*/*P. pastoris* Shuttle Vectors

In order to complete the new well characterized toolbox for protein expression based on the *P. pastoris* wt strain, a set of new platform strains was generated based on precise knock-outs in the wt strain CBS7435, thereby reducing the risk of undesired additional mutations in the genome that are commonly observed during traditional random mutagenesis approaches. Since negative effects of the *KU70* deletion on strain stability and heterologous protein production cannot be excluded yet, the platform strains were made on the basis of the non-mutagenized wt strain rather than on the *KU70* deletion variant. All excision cassettes (flipper-cassettes) were constructed with overlap-extension PCR having 5′ and 3′ flanking regions from *AOX1* (940 bp and 1143 bp, complete cds knock-out), *ARG4* (963 bp and 1502 bp, partial cds knock-out) and *HIS4* (844 and 882 bp, partial cds knock-out) loci for targeted integration. Wild-type *P. pastoris* was successfully transformed with the respective 5302 bp, 6761 bp and 5985 bp amplification products. Primary selection with Zeocin™ ensured the integration of at least one copy of the cassette in the genome. Transformants were cultivated in 250 µl YPD until reaching stationary phase, followed by stamping on both YPD and MM or MD plates. Normal growth on YPD combined with slow growth on MM (*aox1* knock-out strain) or no growth on MD (*arg4*, *his4* knock-out strains) indicated cassette integration in the target locus. In the *AOX1, ARG4* and *HIS4* loci, approximately 2%, 3% and 1% of the excision cassettes were integrated in the target locus resulting in mut^s^, *arg4* auxotrophic and *his4* auxotrophic phenotypes, respectively. Variation in the integration rates might, for example, be caused by differences in the lengths of the homologous sequences used for integration, excision cassette sizes and secondary structures present in the target loci. Since very low amounts of DNA were used in the transformations, most of the positive transformants were expected to have only a single copy of the selection cassette integrated in the genome.

Flipper cassettes should stay integrated in the genome as long as the tightly controllable promoter chosen for *FLP* is not activated, and thus no FLP recombinase is produced. In the case of the *AOX1* promoter, activation is attained by adding methanol to culture media that contain no repressing carbon source such as glucose or glycerol [51]. Theoretically the number of flipper-cassettes in every transformant can vary and every cell has an individual chance of cassette excision. Therefore, we produced clean single-colony streak-outs from each positive transformant colony and initially induced the promoter only for a short time until the first strains had excised the cassette. This approach should increase the chance to obtain Zeocin™ sensitive cells where only one selection marker was integrated and excised. Original Zeocin™ resistant colonies were streaked out on minimal methanol plates. After two days, no Zeocin™ sensitive colonies were found, indicating that insufficient amounts of FLP had been produced to locate the flipper cassette and perform cassette excision. Therefore, the population was further streaked out on fresh minimal methanol plates and incubated for 3 days. In the case of the *AOX1* promoter in the *AOX1* excision cassette, a five-day induction with methanol on MM plates resulted in the excision of the cassette in ∼7% (16/228) of the single colonies tested, thus rendering the cells Zeocin™ sensitive.

To verify correct cassette integration and excision, high quality genomic DNA was extracted from the wild-type strain and from all knock-out strains before and after cassette excision. Target loci were sequenced using primers located both outside and inside the integration sequences (Table S1). The lengths and sequences of the PCR products with all primer combinations were as expected. The sequences of the targeted sites (Figure S3) confirmed the expected total (*AOX1* locus) or partial (*HIS4* and *ARG4* loci) deletions. Only one *FRT* sequence was left in the genome locus.

DNA samples from the *aox1* deletion strain and the auxotrophic strains before and after knock-out cassette excision were subjected to southern blot analysis to verify the expected knock-out and to define the location and number of excision cassettes integrated in the genome. The results and expected fragment sizes are summarized in Table S4. Wild-type strain CBS7435 was used as a control. *Aox1*, *his4, ku70his4* and *aox1his4* deletion strains were digested with at least two separate restriction enzymes each: NdeI and SspI for the *AOX1* locus, and DraI and BglII for the *HIS4* locus. Every probe targeted specifically to either *AOX1* or *HIS4* wild-type locus showed a band of expected size with the wt control strain CBS7435. As expected, the corresponding fragments were not present in the before and after induction strains with the wild-type locus excised. When using a probe targeted to the Zeocin™ resistance cassette, one band of expected size was observed in all before induction strains still including the excision cassette in the target locus. As expected, no bands could be observed with either wild-type starting strain or any of the after induction strains with the excision cassette flipped out of the genome.

These results suggest a successful, targeted, single-copy integration of the cassettes in the expected locus in all loci tested. Both PCR analysis and sequencing of the modified loci and the results from the southern blots verify the expected excision of the flipper- cassette, leaving only one 34 bp *FRT* in the genome.

Also, a series of new *E.coli*/*P. pastoris* shuttle vectors were constructed during this study. GFP was used as a model protein to test the functionality of all vectors. An overview of the vector features and properties are summarized in Table 5. All pPpB1-based vectors (including the *S. cerevisiae ADH1* promoter and terminator to regulate the expression of the Zeocin™ marker gene) were noted to display distinct background growth upon primary selection of positive transformants. Therefore, only the largest colonies from each plate should be chosen for further screening. However these vectors favour multi copy gene integration and copy numbers of up to 60 gene copies per cell have been reached with this group of vectors (personal communication with Andrea Mellitzer). Contrary to the pPpB1 vectors, transformations with pPpT4–based vectors with ILV5 promoter and AOD terminator to regulate the expression of the Zeocin™ marker gene were noted to lead to no or very little background growth and usually low copy numbers (up to 7 copies/cell). Single copy integration was preferred if a low amount of DNA was used for the transformation.

**Table 5:** New shuttle vectors constructed during this study.

Name	Accession#	Promoter^a^	Localization^b^	Linearization	Selection
pPpB1_S	JQ519685	*AOX1*	Intracellular	Blunt	Zeocin™
pPpB1GAP	JQ519686	*GAP1*	Intracellular	Sticky-end	Zeocin™
pPpB1GAP_S	JQ519687	*GAP1*	Intracellular	Blunt	Zeocin™
pPpB1_Alpha_S	JQ519688	*AOX1*	Secreted	Blunt	Zeocin™
pPpT4	JQ519689	*AOX1*	Intracellular	Sticky-end	Zeocin™
pPpT4_S	JQ519690	*AOX1*	Intracellular	Blunt	Zeocin™
pPpT4_Alpha_S	JQ519691	*AOX1*	Secreted	Blunt	Zeocin™
pPpT4GAP_S	JQ519692	*GAP1*	Intracellular	Blunt	Zeocin™
pPpT4GAP_Alpha_S	JQ519693	*GAP1*	Secreted	Blunt	Zeocin™
pPpKan_S	JQ519694	*AOX1*	Intracellular	Blunt	KanMX6
pPpKan_Alpha_S	JQ519695	*AOX1*	Secreted	Blunt	KanMX6
pPpARG4	JQ519696	*AOX1*	Intracellular	Sticky-end	::*ARG4*
pPpHIS4	JQ519697	*AOX1*	Intracellular	Sticky-end	::*HIS4*
pPpGUT1	JQ519698	*AOX1*	Intracellular	Blunt	::*GUT1*

a Promoter to regulate the expression of the gene of interest.

b Localization of the recombinant protein. Vectors aimed for intracellular production can be used for the secretory production by adding a signal sequence.

### Comparison of Growth Rates of *P. pastoris* CBS7435 Derived Platform Strains

The growth rates of all strains used and created during this study are depicted in Table 1. For all shake flask cultivations, buffered minimal media were used to achieve reproducible results. The growth rates of the *P. pastoris ku70* deletion strain in baffled shake flasks was observed to be only 11% (±0,00%), 10% (±0,01%) and 30% (±0,01%) lower than that of the wild-type starting strain CBS7435 in the respective dextrose, glycerol or methanol minimal media. The *aox1*, *his4* and *arg4* deletion strains constructed during this work showed expected growth rates. Since the commonly used *S. cerevisiae ADH1* promoter was chosen to activate the transcription of the *HIS4* gene in the complementation plasmid pPpHIS4, species specific activation and de-repression of yeast promoters [52] could cause the observed lower growth rate of the complemented *his4* deletion strain on glycerol.

## Discussion

To enable quick and efficient mutant construction for target gene knock-out and characterization in *P. pastoris*, a new strain with reduced non-homologous recombination and thus precise and exclusive integration at the targeted sites is required. In filamentous fungi and higher eukaryotic organisms, the integration of foreign DNA has been reported to occur preferentially via the NHEJ pathway joining DNA ends with little or no homology [53], while in *S. cerevisiae* HR has been reported to be dominant and non-homologous recombination only observed if HR was blocked or homologous chromosome unavailable to serve as a template [54]. The repair process is initiated by a Ku70p/Ku80p heterodimer [7]. This study describes the elucidation of *P. pastoris KU70* and *KU80* genes and for the first time shows experimental data of the involvement of the *P. pastoris KU70* homologue in NHEJ.

To date, most of the modified *P. pastoris* strains have been constructed using unspecific mutagenesis methods or excessively long integration sequences. Gene replacement events have previously been described to occur with a frequency of <0.1% when a total of <500 bp homology, short enough for high throughput screening, is used [12]. We have constructed and characterized a *ku70* deletion strain with a specific 215 bp knock-out in the *KU70* locus. Through analyzing the homologous recombination frequencies in this strain using two well-known auxotrophic loci and homologous sequence lengths varying from 50 bp to 1350 bp, we have shown that when using the *ku70* deletion strain, 100% homologous recombination frequencies can be achieved with as little as 650 bp homologous flanking regions on each side of the integration cassette. For the tested sites, a reasonable number of specific disruptants (>85%) was achieved using a minimum flanking sequence length of 250 bp to 650 bp. This is short enough even to be made synthetically using 1 to 3 oligonucleotides for high throughput screening of genomic loci in *ku70* mutant strains. Only low numbers (0–17%, correlating with the size of the integration sequences) of disruptants can be achieved in the wt strain with any flanking sequence length tested. In the well accessible *HIS4* locus of the *ku70* knock-out strain, over one third of the cassettes showed correct integration with a homologous sequence length as short as 100 bp. Furthermore, the differences between the *HIS4* and the *ADE1* loci confirmed the frequently discussed observations that the efficiency of site-specific integration and absolute knock-out efficiency are locus dependent. This has often caused significant troubles in achieving specific knock-out strains of *P. pastoris* previously (unpublished results). The *KU70* locus seems to play an essential role in the NHEJ mechanism of *P. pastoris*. Knocking out *KU70* reduced the fraction of randomly integrated DNA fragments dramatically. To demonstrate the use of the *ku70* mutant strain in the search of new auxotrophies, we successfully disrupted the *P. pastoris GUT1* gene from the glycerol assimilation pathway creating a new *gut1* deletion strain and complemented the locus with the *E. coli*/*P. pastoris* shuttle vector pPpGUT1. In addition to the use as an auxotrophic strain, a *gut1* deletion strain with complemented *KU70* locus might also be useful as a whole cell biocatalysis platform for biotransformations using glycerol as a substrate due to its inability to efficiently utilize glycerol as a carbon source for biomass production.

For most of the transformants where the selection marker was integrated and no auxotrophy was observed, the *HIS4* or *ADE1* disruption cassettes were expected to be randomly integrated in the genome. By sequencing the integration loci of such “false” and undesired transformants we detected no notable homology of the integration site to the 5′ end of the integrated disruption cassette. Cassettes were found in both coding and non-coding regions distributed among all four chromosomes. Only two strains showed integration of the cassette in the same region, thus provoking discussion about an integration “hotspot” with chromatin structure more permissive for integration. However, future studies with large sets of strains are required to confirm these suggestions. In accordance to previous studies [6]–[11] in yeasts, in two of the 14 strains there was also a single nucleotide deletion at the DSB site.

In addition to the role of Ku70p/Ku80p heterodimer in DNA end joining, it has also been shown to play an important role in the regulation of normal DNA end structure in telomeres [55], [56]. Ku deficient *S. cerevisiae* strains have been reported to have growth defects markedly specific to the culture conditions. Especially cultivation at elevated temperatures has been reported to lead to growth arrest [22], [57]. Ku-deficient *M. musculus* and *S. cerevisiae* cells have been reported to have V(D)J recombination defects, display telomeric shortening and high levels of chromosomal aberrations [21], [23]–[25]. However, our results are more consistent with previous studies in *C. glabrata* [26] and *S. macrospora* [20], where no severe defects in the development and growth could be detected in normal growth conditions. The *P. pastoris ku70* mutant strain was observed to grow only 11% (±0,00%) slower than the corresponding wt strain in minimal dextrose media liquid cultures at 28°C, indicating at least that differences exist for *P. pastoris* too. As suggested previously [55], it is possible that the normal telomerase activity suffices and the activity of the Ku complex in establishing a correct terminal DNA structure is not required when the cells are not dividing too rapidly. However, the effects of the KU70 knock-out on protein expression in large-scale cultivations with higher stress levels have not been evaluated so far. Thus, at this stage direct industrial applications without restoring the normal activity of the Ku70p/Ku80p complex by complementing the locus are not recommended. The slower growth rate could also be caused by DNA damage-induced cell cycle arrest and apoptosis [58]. When exposed to UV rays, the *ku70* mutant strain was observed to be more sensitive than the corresponding wt strain. This fact suggests that the *P. pastoris* Ku70 protein could be involved in the DNA-repair processes. However, despite the possible role of the Ku70 protein in DNA-repair, our results suggest that at least in case of overexpression of non-toxic heterologous proteins such as GFP, deleting the normal function of the *P. pastoris* Ku70 protein neither leads to severe genetic instability nor to an increased loss of expression cassettes within reasonable cultivation times corresponding to 200–300 generations, suggesting that the strain is a suitable host for simple lab-scale experiments. Therefore, the *ku70* mutant strain offers a noteworthy choice, for example, for primary functional studies and screening for selectable markers. Due to the decelerated growth and possible DNA-repair defects exposing the strain to increased genomic instability, complementing the KU70 locus or verification of promising results in the wild-type strain background is recommended. This is of particular importance if the strain generated is destined for industrial scale production of heterologous proteins.

In conclusion, in this study we have shown that the *ku70* deletion strain enables the quick construction of precise site directed genomic integrations and thus contributes, for example, to gene function analyses and the identification of target genes to generate new selection systems. In addition, effects of the overexpression of individual genes can be separated from undesired effects caused by random integration in other important loci of the genome. Although no lethality, severe growth retardation or loss of expression cassettes during four rounds of methanol induction was detected, the *ku70* deletion strain was observed to express decelerated growth and have possible defects in DNA-repair processes. Inactivation of *KU70* could also cause unknown metabolic changes. Therefore, when analyzing the specific functions of genes, the normal activity of *KU70* can also be restored after successful gene targeting. The simplicity of locus complementation is aided by the extremely high homologous integration frequency and the minimal deletion in the *KU70* locus.

The observed simplicity of targeted integration in the *ku70* deletion strain together with the wealth of information provided by the *P. pastoris* full genome sequence opens a new era for the creation and testing of designer strains, for applications in synthetic biology and for supplementing the very limited amount of selectable markers available for *P. pastoris*. Especially regarding gene disruption studies, the efforts to find more biosynthetic markers for the production of pharmaceuticals and the co-expression of multiple proteins or multi-subunit proteins are slow and cumbersome due to the problems caused by NHEJ. Basic expression systems for recombinant protein production in *P. pastoris* have been commercially available in the recent past, but diversity of the available tools has still been low. With the help of the new *ku70* deletion strain of this study we identified a *gut1* [59] deletion strain, which shows no growth on glycerol. This deletion can be simply complemented again, offering antibiotic free selection based on specific carbon sources in the growth media. The *ku70* deletion strain provides quick access to new selection markers and highly specific targeted gene disruptions and insertions for foreign pathway construction. In order to provide a new second generation *P. pastoris* platform without unknown undesired mutations in the genome, we also constructed a series of precise new deletion strains based on the fully sequenced *P. pastoris* wt strain CBS7435, obtained from the Dutch strain collection (also deposited as ATCC 76273) and developed a complementary new versatile platform of *E. coli*/*P. pastoris* shuttle vectors enabling the production of a wide variety of proteins with different requirements concerning promoter efficiency, choice of markers, secretion and, due to the special properties of the *ku70* deletion strain, also precise site specific gene integration and knock-out. Furthermore, the *P. pastoris ku70* deletion strain facilitates the functional characterization of the genome and the metabolic routes of this important protein production host, and it will accelerate further developments of this host platform and corresponding metabolic models.

## Supporting Information

Figure S1 **Vector maps of the** ***E. coli*** **/** ***P. pastoris*** **shuttle vectors constructed during this study.** The origins and functions of plasmid components are depicted in Table S2.
(TIF)

Figure S2 **Southern blots confirming the expected knock-out in the** ***KU70 locus*** **.** a) BamHI (columns 2–5) and BglII (columns 6–9) digested DNA of the wt CBS7435 strain (columns 2 and 6), the *ku70* strain before induction (columns 3 and 7) and two strains after induction (columns 4–5 and 8–9) were detected with a probe specific to the knock-out region in the *KU70* locus. A band of the expected size can be observed in the wt strain only (4368 bp for BamHI and 4127 bp for BglII). b) BamHI (columns 12–15) and BglII (columns 19–22) digested DNA of the wt (columns 12 and 19), *ku70* after induction (13–14 and 20–21) and *ku70* before induction (columns 15 and 22) strains detected with Zeocin-specific probe. A band of the expected size can be observed in the before induction strain only (8412 bp for BamHI and 4837 bp for BglII). Lanes 16 and 23 are empty. Five µl of DIG-labelled ladder #II (Roche) was loaded to lanes 1, 17 and 24, and five µl of DIG-labelled ladder #VII to lanes 10, 11, 18 and 25.
(TIFF)

Figure S3 **Knock-out locus sequences.** The sequences of the targeted sites confirmed the expected total (*aox1*) or partial (*ku70*, *his4, arg4*) deletions. Only one *FRT* sequence (underlined) was left in the genome locus.
(TIFF)

Table S1 **Primers used in this study.** AOX1flipper: primers used in the construction and analysis of the *aox1* strain; probe: primers used for the amplification of the southern blot probes; Ku locus: primers used for the construction and analysis of the *ku70* deletion strain; ARG4flipper: primers used for the construction and analysis of the *arg4* strain; HIS4 flipper: primers used for the construction and analysis of the *his4* strain; Targeting: primers used for the construction and analysis of the disruption cassettes to evaluate targeting efficiencies; pPp: primers used for the construction and analysis of the complementation plasmids; GUT1disrupt: primers used for the construction and analysis of the *gut1* strain; seq: sequencing primer.
(DOCX)

Table S2 **The origins and functions of the** ***E. coli*** **/** ***P. pastoris*** **shuttle vector components.**
(DOCX)

Table S3 **Estimation of the copy number of the GFP expression cassette.** Copy numbers were calculated according to absolute (Abs. Q) and relative (Rel. Q) quantification. Q.1 = quantification before methanol induction, Q.2 = quantification after four rounds of cultivation and induction. No significant changes could be detected in the copy numbers of the wt, mut^s^ and Δ*KU70* strains. GFP production is shown as relative fluorescence units (RFU) normalized by OD.
(DOCX)

Table S4 **Southern blot analysis of the knock-out loci** ***AOX1*** **and** ***HIS4.*** Southern blot analysis was performed to verify the expected knock-outs and to define the location and number of the excision cassettes integrated in the targeted genomes. Fragment  =  size of the hybridizing fragment in the analysis. Probes targeted to the coding sequence (cds) and Zeocin™ resistance cassette (zeo) were used to detect the wild-type (wt) locus, the location and number of Zeocin™ resistance cassettes in the before induction strain still carrying the excision cassette in the targeted locus (flipper in) and the expected knock-out and removal of the Zeocin™ resistance cassette in the final strain after FLP recombinase induction (knock-out).
(DOCX)
