# Supplementary figures and images for: Deletion of the Pichia pastoris KU70 Homologue Facilitates Platform Strain Generation for Gene Expression and Synthetic Biology
Source: PLoS One. 2012 Jun 29;7(6):e39720. doi: 10.1371/journal.pone.0039720 (PMC3387205; doi:10.1371/journal.pone.0039720)

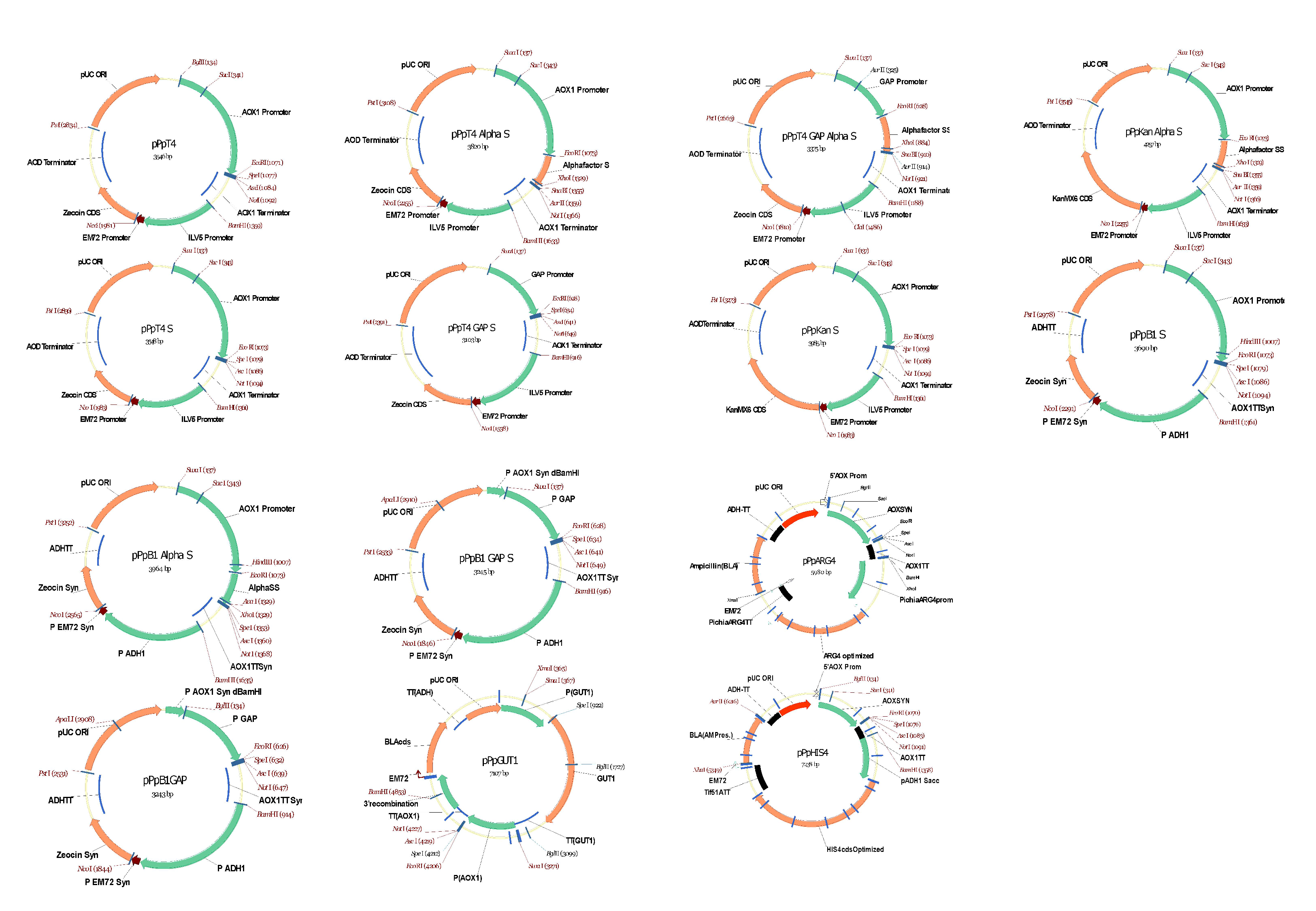

Supplement: Figure S1 — Vector maps of the E. coli / P. pastoris shuttle vectors constructed during this study. The origins and functions of plasmid components are depicted in Table S2. (TIF) [file pone.0039720.s001.tif]

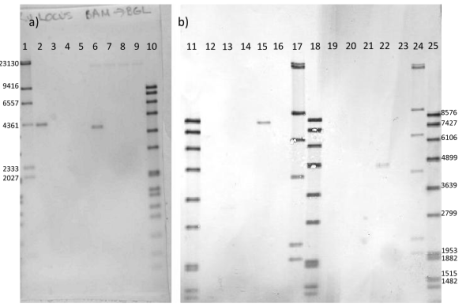

Supplement: Figure S2 — Southern blots confirming the expected knock-out in the KU70 locus . a) BamHI (columns 2–5) and BglII (columns 6–9) digested DNA of the wt CBS7435 strain (columns 2 and 6), the ku70 strain before induction (columns 3 and 7) and two strains after induction (columns 4–5 and 8–9) were detected with a probe specific to the knock-out region in the KU70 locus. A band of the expected size can be observed in the wt strain only (4368 bp for BamHI and 4127 bp for BglII). b) BamHI (columns 12–15) and BglII (columns 19–22) digested DNA of the wt (columns 12 and 19), ku70 after induction (13–14 and 20–21) and ku70 before induction (columns 15 and 22) strains detected with Zeocin-specific probe. A band of the expected size can be observed in the before induction strain only (8412 bp for BamHI and 4837 bp for BglII). Lanes 16 and 23 are empty. Five µl of DIG-labelled ladder #II (Roche) was loaded to lanes 1, 17 and 24, and five µl of DIG-labelled ladder #VII to lanes 10, 11, 18 and 25. (TIFF) [file pone.0039720.s002.tiff]

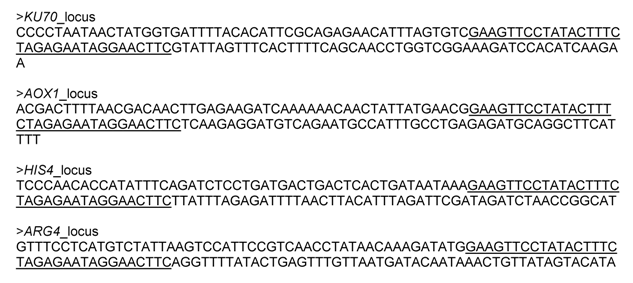

Supplement: Figure S3 — Knock-out locus sequences. The sequences of the targeted sites confirmed the expected total (aox1) or partial (ku70, his4, arg4) deletions. Only one FRT sequence (underlined) was left in the genome locus. (TIFF) [file pone.0039720.s003.tiff]
