# Supplementary material for: Deletion of the Pichia pastoris KU70 Homologue Facilitates Platform Strain Generation for Gene Expression and Synthetic Biology
Source: PLoS One. 2012 Jun 29;7(6):e39720. doi: 10.1371/journal.pone.0039720 (PMC3387205; doi:10.1371/journal.pone.0039720)
Supplement: Table S1 — Primers used in this study. AOX1flipper: primers used in the construction and analysis of the aox1 strain; probe: primers used for the amplification of the southern blot probes; Ku locus: primers used for the construction and analysis of the ku70 deletion strain; ARG4flipper: primers used for the construction and analysis of the arg4 strain; HIS4 flipper: primers used for the construction and analysis of the his4 strain; Targeting: primers used for the construction and analysis of the disruption cassettes to evaluate targeting efficiencies; pPp: primers used for the construction and analysis of the complementation plasmids; GUT1disrupt: primers used for the construction and analysis of the gut1 strain; seq: sequencing primer. (DOCX) [file pone.0039720.s004.docx]

**Table S1. Primers used in this study.**

| Oligo # | Oligo name | Sequence 5’-3’ |
| --- | --- | --- |
| P07614 | AOX1flipper_1F | AGATCTAACATCCAAAGACGAAAGGTTGAATGAAACC |
| P07615 | AOX1flipper_1R | GAAGTTCCTATTCTCTAGAAAGTATAGGAACTTCCGTTTCAATAATTAGTTGTTTTTTG |
| P07616 | AOX1flipper_2F | CTATACTTTCTAGAGAATAGGAACTTCATGCCACAATTTGATATATTATG |
| P07617 | AOX1flipper_2R | CAAGACATTACTGAATAAGCTTACATTATGAAGAGCAGC |
| P07618 | AOX1flipper_3F | GTAATATGCTGCTCTTCATAATGTAAGCTTATTCAGTAATGTCTTGTTTCTTTTG |
| P07619 | AOX1flipper_3R | GAAGTTCCTATTCTCTAGAAAGTATAGGAACTTCCTAAGGTAATCAGATCCAAG |
| P07620 | AOX1flipper_4F | CTATACTTTCTAGAGAATAGGAACTTCTCAAGAGGATGTCAGAATG |
| P07621 | AOX1flipper_4R | GATCTTGAGATAAATTTCACGTTTAAAATCAGCGTACCTTTTTCTCG |
| P07626 | AOX1flipper_1F(short) | AGATCTAACATCCAAAGACG |
| P07627 | AOX1flipper_4R(short) | GATCTTGAGATAAATTTCACG |
| P07628 | AOX1flipper_1FRTFseq | GGTGCACCTGTGCCGAAACG |
| P07629 | AOX1flipper_2FRTRseq | GTTCCGTTATGTGTAATCATCCAAC |
| P07630 | AOX1flipper_3FRTFseq | CATATTGCCTACGCATGTATAGGTG |
| P07631 | AOX1flipper_4FRTRseq | CGAGATAGGCTGATCAGGAG |
| P07636 | AOX1flipper_2RnewCYC | CAAAGGAAAAGGGGCCTGTTTATATGCGTCTATTTATGTAG |
| P07637 | AOX1flipper_CYC1F | ACATAAATAGACGCATATAAACAGGCCCCTTTTCCTTTGTCGATATC |
| P07638 | AOX1flipper_CYC1R | GAAACAAGACATTACTGAAGTCGACAACTAAACTGGAATGTGAGG |
| P07639 | AOX1flipper_3FnewCYC | CTCACATTCCAGTTTAGTTGTCGACTTCAGTAATGTCTTGTTTCTTTTG |
| P08-31 | ZEOprobe_EM72F | GACACTTTATACTTCCGGCTCG |
| P08-32 | ZEOprobe_ZEOR | CTGCTCTTCTGCGACGAAATGC |
| P08-33 | AOX1cdsProbeF | TGCTTCTGATTACGATGACTTCC |
| P08-34 | AOX1cdsProbeR | CAAAACCGGATCTTTGCAAAACCAAT |
| P08-35 | AOX2cdsProbeF | GGTGACGCTAACATTCAAAAGAAG |
| P08-36 | AOX2cdsProbeR | GGAAGTAACATGTCCTTCGTTTC |
| P0879 | Kulocus_UpStrm_fwd | AACATGAAAGTAATATGGAACTCCG |
| P0880 | Kulocus_UpStrm2_fwd | AAGCAAAGGGTTGTATAGGC |
| P0881 | Kulocus_DnStrm_rev | TGAATCAGGCAGTCTGCATTCC |
| P0882 | Kulocus_mutATG_rev | GCTTGCTGACAACACTGTATCTTGCAATGCTTTTTATTATTCTC |
| P0883 | Kulocus_mutATG_fwd | GCAAGATACAGTGTTGTCAGCAAGC |
| P0884 | Kulocus_orf_end_rv | CAACATAGGCAATGTGGTGG |
| P0885 | Kulocus_mid_orf_fwd | CACATTCGCAGAGAACATTTACTTGTC |
| P0886 | Kulocus_mid_orf_rev | GACAACTAAATGTTCTCTGCGAATGTG |
| P0887 | Kulocus_kulink5_rev | CCTTTCGTCTTTGGATGTTAGATCTGACAACTAAATGTTCTCTGC |
| P0888 | Kulocus_kulink3_fwd | CAGAGTACAGAAGATTAAGTGAGACCTTCGTTGTCTTTTACAATCCATGACC |
| P0889 | Kulocus_flipend_rev | CGAAGGTCTCACTTAATCTTCTGTACTCTG |
| P0896 | Kulocus_FRT2-KU_f | CTATACTTTCTAGAGAATAGGAACTTCGTATTAGTTTCACTTTTCAGCAAC |
| P08101 | Kulocus_ko_miss_f | GTTCTTCCTGATAAAGCTCC |
| P08102 | Kulocus_ko_mutATG_R | TGCTTGCTGACAACACTGT |
| P08103 | Kulocus_plLV_f | ATCCTTCAGTAATGTCTTG |
| P08104 | Kulocus_KU_end_f | CCCCCACGACAGTAGACC |
| P08105 | Kulocus_KU_5-2_f | GTAGACATTACTTTGATTCCG |
| P08106 | Kulocus_KU_5_1_r | CCAAATTCAGGTCTTGTAAC |
| P08107 | ADE_seq1 | TTTCATCGTGTTCACCCTG |
| P08108 | ADE_seq2 | CACAGACTGATACCTTTGG |
| P08109 | ARG_seq1 | CTCGATTTTGATAGCATCC |
| P08110 | ARG_seq2 | AGGGACAACTGATCAATACC |
| P08111 | ARG_seq3 | CAACCAGTGAGACCATC |
| P08112 | URA_seq1 | CGACATAATTGATGACTTCAC |
| P08113 | URA_seq2 | CATGGGCAATTGATCC |
| P08114 | HIS_seq1 | GAGTAATTAGAAGAGTCAGCC |
| P08115 | HIS_seq2 | TTCTGGATAGGACGACG |
| P08116 | HIS_seq3 | ATCTTGGCAGCAGTAACG |
| P08117 | HIS_seq4 | TGCTGGGTGTTCCTGC |
| P08119 | Kulocus_KU70+plLV_f | CACATTCGCAGAGAACATTTAGTTGTCGATCCTTCAGTAATGTCTTG |
| P08134 | ARG4flipper_P(AOX)F | GAAGTTCCTATACTTTCTAGAGAATAGGAACTTCAGATCTAACATCCAAAGACGAAAGG |
| P08135 | ARG4flipper_P(AOX)R | CATAATATATCAAATTGTGGCATCGTTTCAATAATTAGTTGTTTTTTGATCTTCTCAAG |
| P08141 | ARG4flipper_5'UTRseqF | GGTTGGATTCATCGTCTTCGTGC |
| P08142 | ARG4flipper_3'UTRseqR | GACAGTTCTATCTACCCGAGGAAACC |
| P08158 | Targeting_ADE_2_fw | GGCACCCTACATAAAGAATC |
| P08159 | Targeting_ADE_3_fw | CCATGTGTCATCGCTTCC |
| P08160 | Targeting_ADzeo1r | GAAGCTATGGTGTGTGGGCCAGTGATGTAACCTCTGACAATGGC |
| P08161 | Targeting_ADzeo1f | GGCCCACACACCATAGCTTC |
| P08162 | Targeting_zeoAD2r | TTGCTCACATGTTGGTCTCC |
| P08163 | Targeting_zeoAD2f | GGAGACCAACATGTGAGCAAAAGGAAGTGCATGGAAAGAGTACAAGAAC |
| P08164 | Targeting_ADE_1_rv | ATGATCATTGTTTACTAATTACC |
| P08165 | Targeting_ADE_3_rv | GCGATTTACCCACTTGG |
| P08172 | ARG4_5‘seqF | GAAAGATGACCGATACTATTGG |
| P08173 | ARG4_3‘seqR | GCTTGTCTGACACATTCACC |
| P08174 | P(AOX)seqR | GAGAAGAGGAGTGGAGGTCC |
| P08183 | HIS4flipper_5H4intR | GAAGTTCCTATTCTCTAGAAAGTATAGGAACTTCTTTATTATCAGTGAGTCAGTCATCAGG |
| P08184 | HIS4flipper_3H4intF | GAAGTTCCTATACTTTCTAGAGAATAGGAACTTCTTATTTAGAGATTTTAACTTAC |
| P08193 | HIS4flipper_5H4intF | CTCCACCAATCAATTCTGGGGATTTGGCTCC |
| P08194 | HIS4flipper_3H4intR | CCTTGACTTTCAGCTGACGTTGGAGTTCG |
| P08195 | HIS4flipper_H4intseqF | GAACAACTGGACTAACACCAGAACCTGC |
| P08196 | HIS4flipper_H4intseqR | CCACATTTCCTACGAACTTGAGTATGGC |
| P08197 | HIS4flipper_H4outseqF | CCAATGAAATTATTCAGCAATCGAGAGC |
| P08198 | HIS4flipper_H4outseqR | CAAATCATCGATTTCACGCTGGTATCC |
| P08341 | pPp_2AMP_ADHTT_R | CATAAGAAATTCGCCCTAGGTTACCAATGCTTAATCAGTGAGG |
| P08342 | pPp_3AMP_ADHTT_F | CACTGATTAAGCATTGGTAACCTAGGGCGAATTTCTTATGATTTATG |
| P08343 | pPp_4PADH_HIS_R | GAAGCAAGGGAAAGGTCATTGTATATGAGATAGTTGATTGTATGC |
| P08344 | pPp_5PADH_HIS_F | CAACTATCTCATATACAATGACCTTTCCCTTGCTTC |
| P08345 | pPp_6HIS_TIFTT_R | GATGTTAACCGGTGCGGCCTTAAATCAAACCAAGCTTCTCC |
| P08346 | pPp_7HIS_TIFTT_F | GAGAAGCTTGGTTTGATTTAAGGCCGCACCGGTTAACATC |
| P08347 | pPp_4PARG_AOXTT_R | GAGCAGGTAAAGCGGTCCTCGAGGGATCCGCACAAACGAAGGTC |
| P08348 | pPp_5AOXTT_PARG_F | GTTTGTGCGGATCCCTCGAGGACCGCTTTACCTGCTCTTG |
| P08349 | pPp_6CDSARG_PARG_R | CTCTCTTCCTGGTTAGACATAGATAGCTGGTAATAAGTTTAGAACAAAAG |
| P08350 | pPp_7PARG_CDSARG_R | CTAAACTTATTACCAGCTATCTATGTCTAACCAGGAAGAGAG |
| P08351 | pPp_8TTARG_CDSARG_R | CAAACTCAGTATAAAACCTATTAGGATTCAAGTTTCTCATTCAAG |
| P08352 | pPp_9CDSARG_TTARG_F | GAATGAGAAACTTGAATCCTAATAGGTTTTATAATGAGTTTGTTAATGATAC |
| P08353 | pPp_10EM72_ARGTT_R1 | ATTATACGAGCCGGAAGTATAAAGTGTCAACACCTGTACCGGTTTACAGAAGG |
| P08354 | pPp_P72_ARGTT_XmaR2 | AAATTCCCGGGTTTAGTCCTCCTTACACCTTGTCGTATTATACGAGCCGGAAGTATAAAG |
| P08355 | pPp_1AMP_Xmal_F | TAAACCCGGGATGAGTATTCAACATTTCCGTGTC |
| P08356 | pPp_1AMP_Xbal_F | TAAATCTAGAATGAGTATTCAACATTTCCGTGTC |
| P08357 | pPp_8TIFTT_EM72_R | GGAAGTATAAAGTGTCAACACCCTGCAGGACTCGAACCTG |
| P08358 | pPp_9TIFTT_EM72_F | GGTTCGAGTCCTGCAGGGTGTTGACACTTTATACTTCC |
| P08359 | pPp_10EM72_Xbal_R | ATATTCTAGATTTAGTCCTCCTTACACCTTG |
| P08478 | pPp_HIScdsSeqR | GACTTGAAGCTCGGTGGACTGTG |
| P08479 | pPp_HIScdsSeqF | CAGCTCTGGAACCAATCATAC |
| P08480 | pPp_ARGcdsSeqR | CAGTGTAGACCTTTGTACCTTC |
| P08481 | pPp_ARGcdsSeqF | CATCACATTTCTGGTGAATGTGTG |
| P08482 | pPp_AMPcdsSeqR | CAAAAAAGCGGTTAGCTCCTTC |
| P08579 | pPp_Seq1amp | CTGGATCTCAACAGCGGTAAG |
| P08580 | pPp_Seq2amp | GTGACACCACGATGCCTGTAG |
| P08581 | pPp_PAOX800F | CTGTTCTAACCCCTACTTG |
| P08582 | pPp_AOXTTseqF | GTGGTAGGGGTTTGGGAAAATC |
| P08583 | pPp_ARGcdsSeqF2 | CTCTTGGTGCTGGAGCACTTG |
| P08584 | pPp_ARGcdsSeqR2 | CTCACCAGTGCTGTAGATG |
| P08585 | pPp_ARGTTseqF | CTGACTGTCGTACGGCCTAG |
| P08586 | pPp_PADHseqF | CATCATCATATCGAAGTTTCACTAC |
| P08587 | pPp_HIScdsSeqF2 | CTTACTCCTGAGGTCATCTATGTC |
| P08588 | pPp_HIScdsSeqF3 | GTTACTCGACGTAAAGGTGATG |
| P08589 | pPp_HIScdsSeqF4 | TTCTCTTACCACAGACCGTCCAG |
| P08697 | pPp_ARG4cds1F | CTAACTAAAGACGAACTAAGTGAG |
| P08698 | pPp_ARG4cds1R | CAATGTATTCACGATCAATTCCATAAG |
| P08699 | pPp_ARG5intRs | GTGTGGAACCTCCTTCCACTTG |
| P08700 | HISprobe1F | CACTAGAAGGAAAGGAGATGCCAAG |
| P08701 | HISprobe1R | CTTTGGCAGGAACACCCAGCATC |
| P08721 | pPp_pUCoriF | CTGCGCGTAATCTGCTGCTTGC |
| P08722 | pPp_PAOXstartF | CTAACATCCAAAGACGAAAGGTTG |
| P08723 | pPp_HIScdsSeqR2 | CTCAATGCCAAGCAACTTCTGTG |
| P08763 | Targeting_ADE_4_rv | GGACAGTTTTTGAGTTCTTG |
| P08764 | Targeting_HI-1000-r | GCAGGATCAAGTGTTCAGG |
| P08765 | Targeting_HI-250-r | CTATAGAGAGATCAATGGCTC |
| P08766 | Targeting_HI-100-r | GGCTTTGTCACCATTTTG |
| P08767 | Targeting_HI-1000-f | CCTAGATTTGGCAGAAAGAG |
| P08768 | Targeting_HI-640-f | GGCTGACTCTTCTAATTACTCG |
| P08769 | Targeting_HI-250-f | CTTGCAGAAGCTAAATCC |
| P08770 | Targeting_HI-100-f | CCAAGCCAGGATACACC |
| P08779 | Targeting_HI-zeo-1-r | GAAGCTATGGTGTGTGGGCCTTAGAAACGTCAATTTTGC |
| P08780 | Targeting_HI-zeo-2-f | GGAGACCAACATGTGAGCAAAAGGCCTCCTCACAAGAAATTG |
| P08781 | Targeting_UR-zeo-1-r | GAAGCTATGGTGTGTGGGCCTTGATATTGATGCTTGACAG |
| P08782 | Targeting_UR-zeo-2-f | GGAGACCAACATGTGAGCAAAAGGAGGTGTCTACAAGATTGCAC |
| P08840 | ARGprobe5intF | CTATTAGAAGGGTTTACGATGAGGAAG |
| P08841 | ARGprobe5intR | CACCAATAGTATCGGTCATCTTTCTC |
| P09076 | Targeting_UpStrm2_fwd | AAGCAAAGGGTTGTATAGGC |
| P09077 | Targeting_DnStrm_rev | TGAATCAGGCAGTCTGCATTCC |
| P09078 | Targeting_UpStrm2_BamHI_fwd | CGCAGGATCCAAGCAAAGGGTTGTATAGGC |
| P09079 | Targeting_DnStrm_BamHI_rv | CTACCCGGGTGAATCAGGCAGTCTGCATTCC |
| P09147 | Targeting_Upstrm_KU70_rev | GGATGTCGTATTGCTTGCTGAC |
| P09148 | Targeting_Dnstrm_KU70_Mitte_fw | GGTGGATCAATTACGAAAATACG |
| P09149 | Targeting_Dnstrm_KU70_Ende_fw | CAGATGATGCACAGAAACAACG |
| P09309 | ARG4flipper_ARG4locusR3 | CTCAGGAGATCCGCATCAGACGAAG |
| P09310 | ARG4flipper_ARG4locusR2 | GAGACTCTGTCGACAGTTCTATCTAC |
| P09311 | ARG4flipper_ARG4locusR1 | GTACAACGAAGTGCTCTTGTCATACC |
| P09312 | ARG4flipper_ARGlocusF3 | CCTGCTCTTGGAGACGTTTACTG |
| P09313 | ARG4flipper_ARG4locusF2 | GCTAATTTGGCTGCTGAGAAGGACG |
| P09314 | ARG4flipper_ARG4locusF1 | GAATAGTTGAACCCTTGAACGAAGAGG |
| P09522 | pPp_GUTseq1 | GATCTGGTGCGAAGCAACAG |
| P09523 | pPp_GUTseq2 | GGTACTTTGCCGACTCCTC |
| P09524 | pPp_GUTseq3 | GTCTTGCTGCTTGTTTAGTCAC |
| P09525 | pPp_GUTseq4 | CAGAACCAACTTCATGAACATTG |
| P09526 | pPp_GUTseq5 | CGTCGGACCATTGGCTTC |
| P09527 | pPp_GUTseq6 | CTTGGCTGCAGGGAACAC |
| P09528 | pPp_GUTseq7 | CTTTCTACTAGATATTCTGGAACTG |
| P09529 | pPp_GUTseq8 | CAACAAGCTCCGCATTACAC |
| P09530 | pPp_GUTseq9 | GATCAGCCTACTTCGCAG |
| P09531 | pPp_GUTseq10 | CCTTAGGATCCTTTTCTCTTCTAC |
| P09532 | pPp_GUTseq11 | CCAGAAACGCTGGTGAAAG |
| P09533 | pPp_GUTseq12 | CACGATGCCTGTAGCAATG |
| P09534 | pPp_GUTseq13 | CATGAGGTCGCTCTTATTGAC |
| P09535 | pPp_GUTseq14 | GGTTGGACTCAAGACGATAG |
| P09536 | GUT1disrupt_GUTlocus_1F | CTTTTGCTGGCCTTTTGCTCACAATACCGAAAGGTTAAACAACTTCG |
| P09537 | GUT1disrupt_GUTlocus_1R | CAACCTTT CGTCTTTGGATGTTATTTAAATTGCCAGAGCTGTCACATACTTG |
| P09540 | pPp_3rec_3R | ATTAGTGAGACCTTCGTTTGTGCGCGTTGTATATTCGGTTGGTTTTCC |
| P09541 | pPp_3recPml_3R | TATTAACACGTGCAAGTTGAACTAAAGAACGGAAC |
| P09542 | pPp_AmpPml_4F | AATTACACGTGTTGACACTTTATACTTCCGGCTCG |
| P09543 | pPp_pUCORl_4R | GAAGTTGTTTACCTTTCGGTATTGTGAGCAAAAGGCCAGCAAAAG |
| P10038 | GUT1disrupt_GUTout3prR2 | GGTTCTTGATGAAGCTTATATCG |
| P10039 | P(AOX)_124R | GAGAAGAGGAGTGGAGGTC |
| P10040 | GUT1disrupt_GUTout3prR1 | ATGAAGTTAGTAAGGTTCTTGATGAAGC |
| P10041 | GUT1disrupt_GUTout5prF1 | CGCTCCTGACTGTTTCAAGTC |
| P10042 | GUT1disrupt_GUTout5prF2 | GCATTGTTCTTTGAAATCGAAATTGG |
| P10529 | KuProbeF | CAATCCATGACCAAAAAATCCAAG |
| P10530 | KuProbeR | CAATTTTGGGTGGCAGCTG |
| - | Adaptor primer 1 | GTAATACGACTCACTATAGGGC |
| - | Adaptor primer 2 | ACTATAGGGCACGCGTGGT |
| P11093 | Gene specific primer 1 | CCAAACCTTTAGTACGGGTAATTAACG |
| P11095 | Gene specific primer 2 | GTCCTCCACGAAGTCCCGG |
| P11075 | ARG4flipper_ARG5intF1 | ATCAAAATTGAAGATGACTTACTTGATAACATCC |
| P11076 | ARG4flipper_ARG3intR1 | TGAGTCATTACCGGAAGCTAGAAC |
| P11077 | ARG4flipper_ARG5intF2 | CTTATTACCAGCTATCTATACTCGAATCAAGAAGAAGGAC |
| P11078 | ARG4flipper_ARG3intR2 | ATCAAAATTGAAGATGACTTACTTGATAACATCC |
| P11079 | ARG4flipper_ARG5seq1F | CGTACGACAGTCAGTTAGTAG |
| P11080 | ARG4flipper_ARG5seq1R | TACCCTGATTAGATAATACAATAACCAAC |
| P11081 | ARG4flipper_ARG5seq2F | CAAGTTGGCAGATGCTTATTCTAC |
| P11082 | ARG4flipper_ARG3seq2R | CTAGGCCGTACGACAGTCAG |
| P11083 | ARG4flipper_ARG5intR1 | GAAGTTCCTATTCTCTAGAAAGTATAGGAACTTCAGGTTTTATACTGAGTTTGTTAATGATAC |
| P11084 | ARG4flipper_ARG3intF1 | GAAGTTCCTATACTTTCTAGAGAATAGGAACTTCAGATAGCTGGTAATAAGTTTAGAACAAAAG |
| P11085 | ARG4flipper_ARG5intR2 | GAAGTTCCTATTCTCTAGAAAGTATAGGAACTTCCATATCTTTGTTATAGGTTGAC |
| P11086 | ARG4flipper_ARG3intF2 | GAAGTTCCTATACTTTCTAGAGAATAGGAACTTCAGGTTTTATACTGAGTTTGTTAATGATACAATAAAC |

Naming of the primers is as follows: AOX1flipper_, primers used in the construction and analysis of the *aox1* strain; probe, primers used in the amplification of the southern blot –probes; Kulocus_, primers used in the construction and analysis of the *ku70* deletion strain; ARG4flipper_, primers used in the construction and analysis of the *arg4* strain; HIS4 flipper_, primers used in the construction and analysis of the *his4* strain; Targeting_, primers used in the construction and analysis of the disruption cassettes to evaluate targeting efficiencies; pPp_, primers used in the construction and analysis of the complementation plasmids; GUT1disrupt_, primers used in the construction and analysis of the *gut1* strain; seq, sequencing primer
