# Supplementary material for: Deletion of the Pichia pastoris KU70 Homologue Facilitates Platform Strain Generation for Gene Expression and Synthetic Biology
Source: PLoS One. 2012 Jun 29;7(6):e39720. doi: 10.1371/journal.pone.0039720 (PMC3387205; doi:10.1371/journal.pone.0039720)
Supplement: Table S2 — The origins and functions of the E. coli / P. pastoris shuttle vector components. (DOCX) [file pone.0039720.s005.docx]

**Table S2.** **The origins and functions of the *E. coli*/*P. pastoris* shuttle vector components.**

| **Element** | **Origin** | **Function** |
| --- | --- | --- |
| P_AOX1Syn_  dBamHI | Synthetic | Part of *Pichia pastoris* *AOX1* promoter |
| P_AOX1Syn | Synthetic | *AOX1* promoter for methanol induced expression of the target gene in *Pichia pastoris* |
| P_GAP | CBS7435, *Pichia pastoris* strain | *GAP1* promoter for constitutive expression of the target gene in *Pichia pastoris* |
| P_ADH1 | BY4741, *Saccharomyces cerevisiae* strain | *ADH1* promoter for expression of respective antibiotic resistance gene in *Pichia pastoris* |
| P_ILV5 | CBS7435, *Pichia pastoris* strain | *ILV5* promoter for expression of respective antibiotic resistance gene in *Pichia pastoris* |
| P_TEF1 | BY4741, *Saccharomyces cerevisiae* strain | *TEF1* promoter for expression of respective antibiotic resistance gene in *Pichia pastoris* |
| P_EM72 Syn | Synthetic consensus sequence *E. coli* promoter | Constitutive promoter for expression of respective antibiotic resistance gene in *E. coli* |
| P_ARG4 | CBS7435, *Pichia pastoris* strain | *ARG4* promoter for expression of *ARG4* gene |
| AOX1TTSyn | Synthetic | *AOX1* terminator for target gene transcription termination in *Pichia pastoris*. |
| TIF51ATT | BY4741, *Saccharomyces cerevisiae* strain | *TIF51A* terminator for target gene transcription termination in *Pichia pastoris*. |
| ADH1TT | BY4741, *Saccharomyces cerevisiae* strain | *ADH1* terminator for target gene transcription termination in *Pichia pastoris*. |
| AODTT | CBS7435, *Pichia pastoris* strain | *AOD* terminator for target gene transcription termination in *Pichia pastoris*. |
| CYC1TT | BY4741, *Saccharomyces cerevisiae* strain | *CYC1* terminator for target gene transcription termination in *Pichia pastoris*. |
| ARG4TT | CBS7435, *Pichia pastoris* strain | *ARG4* terminator for target gene transcription termination in *Pichia pastoris*. |
| Zeocin Syn | Synthetic, *Sh ble* codon optimized :Leto, Entelechon®, mixed codon usage (*E. coli, P. pastoris*) | Zeocin resistance, selection marker in *E.coli* and *Pichia pastoris* |
| KanMX6 | KanMX6 (60, 61) (ka et al., 1981;Wach et al., 1994) | Kanamycin and Geneticin resistance in *E. coli* and *Pichia pastoris*, respectively; selection marker |
| BLAcds | *β-lactamase* gene from pUC8 | Ampicillin resistance in *E. coli,* selection marker |
| pUC origin | pBR322 | pUC replication origin for *E.Coli* |
| Alphafactor | Synthetic, codon optimized :Leto, Entelechon®, based on *Saccharomyces cerevisiae* prepro alpha-factor (MF alpha-2) | Secretion signal sequence |
| ARG4_optimized | Synthetic, codon optimized: Gene Designer, Leto (Entelechon®); *Pichia pastoris*, *Yarrowia lipolytica*, *Schizosaccharomyces pombe* average codon usage leaving out rarest codons from all 3 organisms | *Pichia* wild-type gene coding for  *argininosuccinate lyase*, selection marker |
| HIS4_optimized | Synthetic, codon optimized: Gene Designer, Leto (Entelechon®); Synthetic *Pichia pastoris*, *Yarrowia lipolytica*, *Schizosaccharomyces pombe* average codon usage leaving out rarest codons from all 3 organisms | *Pichia* wild-type gene coding for  trifunctional *HIS4*, selection marker |
| KU70 | CBS7435, *Pichia pastoris* strain | Homologue of *S. cerevisiae* *HDF1* in *Pichia pastoris* |
| FRT | Synthetic minimal FRT site 1988)(62) | FLP recombinase target sequence |
| *FLP recombinase* | BY4741, *Saccharomyces cerevisiae* strain | Site-specific recombinase, breakage and joining of four DNA strands between two target sequences |
| *PDI704* | CBS704, *Pichia pastoris* strain | Protein Disulfide Isomerase, Chaperone |
| Syn*PDI* | Synthetic, based on PDI from *Pichia pastoris* strain X-33, codon optimized with Leto 1.0, Entelechon® | Protein Disulfide Isomerase, Chaperone |

**Supplementary References**

60. Oka Y, Ishida H, Morioka M, Numasaki Y, Yamafuji T et al. (1981) Combimicins new kanamycin derivatives bioconverted by some *Micromonosporas*. Journal of Antibiotics 34: 777-781.

61. Wach A, Brachat A, Pöhlmann R, Philippsen P (1994) New heterologous modules for classical or PCR-based gene disruptions in *Saccharomyces cerevisiae*. Yeast 10: 1793-808.

62. Umlauf SW, Cox MM (1988) The functional significance of DNA sequence structure in a site-specific genetic recombination reaction. The EMBO journal 7: 1845-1852.
