# Supplementary material for: Deletion of the Pichia pastoris KU70 Homologue Facilitates Platform Strain Generation for Gene Expression and Synthetic Biology
Source: PLoS One. 2012 Jun 29;7(6):e39720. doi: 10.1371/journal.pone.0039720 (PMC3387205; doi:10.1371/journal.pone.0039720)
Supplement: Table S3 — Estimation of the copy number of the GFP expression cassette. Copy numbers were calculated according to absolute (Abs. Q) and relative (Rel. Q) quantification. Q.1 = quantification before methanol induction, Q.2 = quantification after four rounds of cultivation and induction. No significant changes could be detected in the copy numbers of the wt, muts and ΔKU70 strains. GFP production is shown as relative fluorescence units (RFU) normalized by OD. (DOCX) [file pone.0039720.s006.docx]

**Table S3.** **Estimation of the copy number of the GFP expression cassette.**

| **Strain** | **Abs Q.1** | **Rel. Q.1** | **Copies** | **GFP(RFU)*OD-1** | **Abs Q.2** | **Rel. Q.2** | **Copies** |
| --- | --- | --- | --- | --- | --- | --- | --- |
| CBS7435wt | 4 | 4 | 4 | 2966 | 4,7 | 4,3 | 4-5 |
| CBS7435mut^s^ | 4,8 | 5 | ~5 | 3876 | 4,9 | 4,4 | 4-5 |
| ∆*KU70*/1 | 4,4 | 4,6 | 4-5 | 1763 | 4,7 | 4,5 | 4-5 |
| ∆*KU70*/2 | 7,5 | 7,5 | 7-8 | 3046 | 7,2 | 6,7 | ~7 |

Copy number calculation according to absolute (Abs. Q) and relative (Rel. Q) quantification. Q.1 = quantification before methanol induction, Q.2 = quantification after four rounds of cultivation and induction. No significant changes could be detected in the copy numbers of the wt, mut^s^ and ∆KU70 strains. GFP production is shown as relative fluorescence units (RFU) normalized by OD.
