# Supplementary material for: Deletion of the Pichia pastoris KU70 Homologue Facilitates Platform Strain Generation for Gene Expression and Synthetic Biology
Source: PLoS One. 2012 Jun 29;7(6):e39720. doi: 10.1371/journal.pone.0039720 (PMC3387205; doi:10.1371/journal.pone.0039720)
Supplement: Table S4 — Southern blot analysis of the knock-out loci AOX1 and HIS4. Southern blot analysis was performed to verify the expected knock-outs and to define the location and number of the excision cassettes integrated in the targeted genomes. Fragment = size of the hybridizing fragment in the analysis. Probes targeted to the coding sequence (cds) and Zeocin™ resistance cassette (zeo) were used to detect the wild-type (wt) locus, the location and number of Zeocin™ resistance cassettes in the before induction strain still carrying the excision cassette in the targeted locus (flipper in) and the expected knock-out and removal of the Zeocin™ resistance cassette in the final strain after FLP recombinase induction (knock-out). (DOCX) [file pone.0039720.s007.docx]

**Table S4.** **Southern blot analysis of the knock-out loci *AOX1* and *HIS4.***

| **Locus** | **Enzyme** | **Probe** | **Fragment/wt** | **Fragment/flipper in** | **Fragment/knock-out** |
| --- | --- | --- | --- | --- | --- |
| *AOX1* | NdeI | cds | 9000bp | - | - |
| *AOX1* | NdeI | zeo | - | 6786bp | - |
| *AOX1* | SspI | cds | 5520bp | - | - |
| *AOX1* | SspI | zeo | - | 963bp | - |
| *HIS4* | BglII | cds | 2650bp | - | - |
| *HIS4* | DraI | zeo | - | 2283bp | - |

Southern blot analysis was performed to verify the expected knock-outs and to define the location and number of the excision cassettes integrated in the targeted genomes. Fragment = size of the hybridizing fragment in the analysis. Probes targeted to the coding sequence (cds) and Zeocin^TM^ resistance cassette (zeo) were used to detect the wild-type (wt) locus, the location and number of Zeocin^TM^ resistance cassettes in the before induction strain still carrying the excision cassette in the targeted locus (flipper in) and the expected knock-out and removal of the Zeocin^TM^ resistance cassette in the final strain after FLP recombinase induction (knock-out).
